# Glaswolle, Halbwertszeit < 40 Tage (faserförmige und granuläre Bestandteile): MAK-Begründung

**DOI:** 10.34865/mb0244gwd10_4or

**Published:** 2025-12-22

**Authors:** Andrea Hartwig, MAK Commission

**Affiliations:** 1 Institut für Angewandte Biowissenschaften. Abteilung Lebensmittelchemie und Toxikologie. Karlsruher Institut für Technologie (KIT) Adenauerring 20a, Geb. 50.41 76131 Karlsruhe Deutschland; 2 Ständige Senatskommission zur Prüfung gesundheitsschädlicher Arbeitsstoffe. Deutsche Forschungsgemeinschaft, Kennedyallee 40, 53175 Bonn, Deutschland. Weitere Informationen: Ständige Senatskommission zur Prüfung gesundheitsschädlicher Arbeitsstoffe | DFG

**Keywords:** Glaswolle, Halbwertszeit < 40 Tage (faserförmige und granuläre Bestandteile), Lunge, Entzündung, Lungentumoren, MAK-Wert, maximale Arbeitsplatzkonzentration, Spitzenbegrenzung, Analogiebetrachtung, Luft, air

## Abstract

The German Senate Commission for the Investigation of Health Hazards of Chemical Compounds in the Work Area (MAK Commission) summarized and evaluated the data for glass wool WHO fibres with a half-life in rats of less than 40 days after intratracheal administration (glass wool t_1/2_ < 40 d) to derive an occupational exposure limit value (maximum concentration at the workplace, MAK value) considering all toxicological end points. In the absence of inhalation studies for these glass wools, studies that investigated the more persistent glass wools, MMVF10 and MMVF11, are used. A two-year inhalation study in rats showed slight irritation in the lungs at 3.1 mg MMVF10/m^3^ and 4.8 mg MMVF11/m^3^, respectively. Two approaches were used to calculate the MAK value: the first applied assessment factors to account for the differences between rats and humans, resulting in a MAK value of 0.1 mg/m^3^ for the respirable fraction (R fraction) of glass wool t_1/2_ < 40 d. The second method calculated the human equivalent concentration, taking into account differences between rats and humans in terms of lifetime exposure, respiratory volume, clearance, lung surface and deposition fraction. The average human equivalent concentration from the two MMVF studies confirmed the MAK value of 0.1 mg/m^3^ R. The MAK value is valid for fibrous dusts and granular components formed from them. Peak Limitation Category II with an excursion factor 8 has been set. It can be assumed that carcinogenic effects induced by fibre toxicity will not occur if the MAK value is observed and glass wool t_1/2_ < 40 d has been classified in Carcinogen Category 4. MMVF10 was not mutagenic in the lungs of BigBlue rats after intratracheal instillation. Studies in germ cells are not available. In the absence of developmental toxicity studies, the substance has been assigned to Pregnancy Risk Group D. Dermal absorption is not expected to contribute significantly to systemic toxicity. There are no data for respiratory or skin sensitization.

**Table:** 

**MAK-Wert (2024)**	**0,1 mg/m^3^ A**
**Spitzenbegrenzung (2024)**	**Kategorie II, Überschreitungsfaktor 8**
	
**Hautresorption**	**–**
**Sensibilisierende Wirkung**	**–**
**Krebserzeugende Wirkung (2024)**	**Kategorie 4**
**Fruchtschädigende Wirkung (2024)**	**Gruppe D **
**Keimzellmutagene Wirkung**	**–**
	
**BAT-Wert**	**–**
	
Molmasse	–
Schmelzpunkt	ca. 800 °C (ECHA 2016)
Dichte	2,5 g/cm^3^ (Nielsen und Koponen 2018)
Löslichkeit	k. A. zu Glaswollen mit Halbwertszeit < 40 Tage Faser X607 (Halbwertszeit 46 Tage): Lösungsgeschwindigkeitskonstante k_dis_ = 990 ng/cm^2^/h (Hesterberg et al. 1998)
	
Verwendung	Isoliermaterial, Gebäudedämmung (Schaeffer und Langfeld 2014), Filtration (Lunn et al. 2009)

In dieser Begründung wird ein MAK-Wert abgeleitet für Glaswollen, deren Faserzahl-Halbwertszeit (HWZ) in der Lunge nach intratrachealer Gabe an Ratten unter 40 Tagen liegt, im Folgenden als Glaswollen (HWZ < 40 d) bezeichnet. Diese Glaswollen werden in der TRGS 905 aufgrund ihrer reduzierten Biobeständigkeit als nicht kanzerogen eingeordnet (Abschnitt 3.1.1; AGS 2021).

Glaswollen (HWZ < 40 d) besitzen keine spezielle CAS-Nr., die CAS-Nr. 65997-17-3 bezeichnet Glaswolle allgemein und damit auch Glaswollen mit längerer Halbwertszeit.

Eingesetzt werden bei der intratrachealen Gabe zur Halbwertszeit-Bestimmung WHO-Fasern (Durchmesser < 3 µm, Länge > 5 µm, Längen/Durchmesser-Verhältnis > 3:1) (siehe Abschnitt 3.1.1).

Da keine ausreichenden Inhalationsstudien mit Glaswollen (HWZ < 40 d) vorliegen, werden Daten mit Glaswollen herangezogen, deren Halbwertszeit nach intratrachealer Gabe über 40 Tagen liegt. Gut dokumentierte Inhalationsstudien liegen mit den gut charakterisierten Glaswollen **MMVF10** und **MMVF11** vor. Ebenfalls werden Studien zu **SG Glaswollefasern, X607-Fasern** und der **0,45-µm-MD-Faser** betrachtet.

Die Fasern **MMVF10**, **MMVF10.1** und **MMVF10a** wurden aus verschiedenen Chargen derselben Firma bezogen. Aerosole von MMVF10.1 in der subchronischen Studie (30 mg/m^3^) und von MMVF10a in der chronischen Studie waren in Bezug auf Fasern pro Kubikzentimeter (sowohl WHO-Fasern als auch Fasern mit einer Länge von > 20 µm), Massenkonzentration und geometrische Mittelwerte ähnlich. Jedoch waren die Lungenbelastungen nach 13 Wochen Exposition für diese beiden Fasern unterschiedlich: Im Vergleich zu MMVF10.1 waren die Lungenbelastungen bei MMVF10a für WHO-Fasern doppelt so hoch und für Fasern > 20 µm fünfmal so hoch. Dies galt sowohl für die Faserzahl pro Lunge als auch pro Milligramm Lunge (Trockengewicht). Bei **MMVF10**, **MMVF10.1** und **MMVF10a** aus verschiedenen Chargen derselben Firma betrugen die Lösungsgeschwindigkeitskonstanten k_dis_ = 300–500 ng/cm^2^/h. Die Unterschiede in den Lungenbelastungen nach 13 Wochen in den beiden Studien könnten mit dem um 30 % höheren Alter und Körpergewicht der Hamster in der Studie mit MMVF10a im Vergleich zur Studie mit MMVF10.1 zusammenhängen (Hesterberg et al. 1999).

Glaswollen mit hohem Calciumoxid-Anteil, sehr niedrigem Boroxid-Anteil und ohne oder minimalem Aluminiumoxid-Anteil zeigen eine gute Löslichkeit (Bellmann et al. 1990). Ein hoher Gehalt an Aluminium wirkt als Netzwerkwandler in Glas und führt dadurch ebenfalls zu guter Löslichkeit von Glaswolle (Guldberg et al. 2000).

Für die Herstellung moderner Glaswollen wird bis zu 80 % recyceltes Glas (Flaschen- und Fensterglas) eingesetzt (Umweltbundesamt 2019).

## 1 Allgemeiner Wirkungscharakter

Die biobeständigeren Glaswollen **MMVF10** und **MMVF11** bewirken nach zweijähriger inhalativer Exposition von männlichen Ratten gegen 3,1 bzw. 4,8 mg/m^3^ eine Makrophagenzunahme in der Lunge, eine Zunahme des relativen Lungengewichts und eine eingeschränkte Lungenclearance. Der Schweregrad der Effekte in der Lunge lässt auf frühe entzündliche Prozesse schließen. Bei 27,8 bzw. 15,8 mg/m^3^ wird eine Zunahme an Adenomen in der Lunge beobachtet.

Es liegen keine Daten zur fruchtschädigenden, keimzellmutagenen und sensibilisierenden Wirkung von Glaswolle (HWZ < 40 d) vor.

## 2 Wirkungsmechanismus

Unter Biobeständigkeit wird bei Fasern die Beständigkeit (Funktion der Biolöslichkeit und der mechanischen Stabilität) verstanden, unter Biopersistenz die Retention der Fasern im Respirationstrakt, die sich aus Biobeständigkeit und Makrophagenclearance ergibt. Durch bronchiale Clearance, Wanderung zu den Lymphknoten oder anderen Orten im Körper, durch Auflösung oder Zerbrechen/Zerfall von Fasern wird die Verweildauer in der Lunge beeinflusst. Die Biolöslichkeit von Fasern ist abhängig von der chemischen Zusammensetzung, der Struktur sowie von Faserdurchmesser und -länge (siehe Abschnitt 3.1; Pott et al. 1991).

Die Zielorgane beim Menschen nach Inhalation von Fasern sind Lunge, Kehlkopf, Pleura und Peritoneum. Bei einer langen Halbwertszeit und wiederholter Aufnahme von Fasern kann es hier zu Entzündungsreaktionen, Fibrosen und Tumoren kommen. Voraussetzungen für diesen Effekt sind bestimmte Abmessungen sowie eine hohe Biobeständigkeit von Fasern. WHO-Fasern mit einer Länge von mehr als 5 µm, einem Durchmesser von weniger als 3 µm und einem Länge-zu-Durchmesser-Verhältnis von mehr als 3:1 können nach dem Einatmen die tiefen Lungenbereiche (Alveolen und Bronchiolen) erreichen (Hartwig und MAK Commission 2018).

Von den biologischen Wirkungen des Asbests ist bekannt, dass nach einer Jahre bis Jahrzehnte dauernden Latenz zwei Formen bösartiger Tumoren, nämlich Karzinome der Lunge und nach noch längerer Latenzzeit Mesotheliome der Pleura und des Peritoneums, entstehen können (Henschler 1981).

Glaswolle (HWZ < 40 d) kann, wie auch andere Fasern, zu entzündlichen Veränderungen in der Lunge führen. Unterhalb eines Grenzwertes für Glaswolle mit geringer Biobeständigkeit, der die entzündlichen Veränderungen in der Lunge verhindert, ist auch keine kanzerogene Wirkung zu erwarten. Der Wirkungsmechanismus der Toxizität und der Kanzerogenese von biobeständigeren Fasern ist bei Hartwig et al. (2018) beschrieben.

Für Mesotheliome, die an der Pleura oder dem Peritoneum nach Belastung mit biobeständigen Fasern bei Menschen auftreten, gilt der Hamster als sensitiv. Auch nach 78 Wochen inhalativer Exposition gegen **MMVF10a** wurden keine Mesotheliome beim Hamster beobachtet (McConnell et al. 1999). Damit ist die vom Asbest bekannte Fasertoxizität nicht aufgetreten, obwohl die Halbwertszeit der Glaswolle MMVF10a bei deutlich mehr als 40 Tagen liegt. Es ist aber auch bekannt, dass Hamster auch nach hoher Belastung mit granulären biobeständigen Stäuben (GBS) keine Lungentumore ausbilden.

Nach intraperitonealer Verabreichung scheint eine anhaltende Entzündung und Zellproliferation innerhalb fibrotischer Noduli die Ursache für die Entwicklung eines Mesothelioms zu sein, das von diesen Knoten ausgeht. Die Inzidenz von Mesotheliomen korrelierte stark mit der Inzidenz von intraabdominalen Noduli und Massen an verschiedenen Stellen. Und die Inzidenz von abdominellen Noduli und Massen korrelierte stark mit der Anzahl der Tiere mit Aszites (Grimm et al. 2002).

## 3 Toxikokinetik und Metabolismus

### 3.1 Aufnahme, Verteilung, Ausscheidung

#### 3.1.1 Intratracheale Aufnahme

Die Biobeständigkeit von Glaswolle kann als Halbwertszeit nach intratrachealer Gabe an Ratten angegeben werden (AGS 2021).

Je 35 weiblichen Wistar-Ratten wurden 2 mg der Glaswollen **B-1M**, **B-2L** oder **B-3L** in 0,4 ml physiologischer Kochsalzlösung intratracheal instilliert. Die Proben wurden vor der Gabe durch Schneiden und Mahlen zerkleinert, um inhalierbare Faserbruchstücke zu erhalten. Nach einem Tag, einem, sechs, zwölf und 24 Monaten wurden die Lungen von je sechs Tieren pro Gruppe entnommen und nach der Trocknung bei 300 °C verascht. Nach der Suspendierung in 1N Salzsäure mithilfe von Ultraschall konnten die Fasern auf Filtern mit einer Porengröße von 0,4 µm abgeschieden werden. Die Bestimmung der Faseranzahl pro Lunge und Fasergröße sowie des Ausgangsmaterials erfolgte mit dem Rasterelektronenmikroskop (REM). Es wurden die in Tabelle 1 dargestellten Halbwertszeiten berechnet. Der Massenanteil und die Zahl kritischer Fasern und die Fasergrößenverteilung der **B-2L**- und der **B-3L**-Fasern waren vor der Applikation sehr ähnlich. Die unterschiedlichen Halbwertszeiten werden daher auf die unterschiedliche Biobeständigkeit zurückgeführt. Die chemische Zusammensetzung ist in Tabelle 2 gezeigt (Bellmann et al. 1990; Pott et al. 1990). Beim Lösen der Glaswolle in der Lunge werden die einzelnen Kationen freigesetzt. Zur Bestimmung der Zusammensetzung werden aufgrund der Analysenmethode die Oxide der Ionen angegeben.

**Tab. 1:** Halbwertszeiten nach intratrachealer Gabe der Fasern B-1M, B-2L, B-3L (Bellmann et al. 1990)

**Faser **	**Mittlere Halbwertszeiten [Tage] für Faserzahl bzw. Partikelmasse**		
	**Faser**	**Faser**	**Partikel**
	**L/D > 3:1, D < 3 µm**	**L/D > 5:1, D < 2 µm**	
B-1M	104	107	116
B-2L	39	38	90
B-3L	240	238	467

D: Durchmesser; L: Länge

**Tab. 2:** Prozentuale chemische Zusammensetzung von Fasern, die intratracheal instilliert wurden (Pott et al. 1990)

**Faser**	**SiO_2_**	**B_2_O_3_**	**Na_2_O**	**Al_2_O_3_**	**FeO + Fe_2_O_3_**	**BaO**	**ZnO**	**K_2_O**	**CaO**	**MgO**
B-1	60,7	3,3	15,4	–	0,2	–	–	0,7	16,5	3,2
B-2	60,7	3,3	15,4	–	0,2	–	–	0,7	16,5	3,2
B-3	58,5	11,0	9,8	5,8	0,1	5,0	3,9	2,9	3,0	–
M475	57,9	10,7	10,1	5,8	0,1	5,0	3,9	2,9	3,0 (CaO + MgO)	

##### Bestimmung der Halbwertszeit nach intratrachealer Instillation

Die Einstufung der Kanzerogenität von WHO-Fasern kann durch Bestimmung der In-vivo-Biobeständigkeit nach intratrachealer Instillation erfolgen. WHO-Fasern werden in die Kategorie 2 der krebserzeugenden Stoffe eingestuft, wenn nach intratrachealer Instillation von 4 × 0,5 mg Glaswolle in einer Suspension eine Halbwertszeit in der Lunge von mehr als 40 Tagen ermittelt wurde. Die WHO-Fraktion der instillierten Faserprobe sollte einen mittleren Durchmesser von 0,6 µm oder mehr aufweisen. Faserproben mit kleinerem Durchmesser können geprüft werden, falls dies mit dem geringeren Durchmesser des Ausgangsmaterials begründet werden kann. Die Halbwertszeit sollte mit nichtlinearer exponentieller Regression berechnet werden. Falls die Elimination biphasisch verläuft, ist die Halbwertszeit der langsamen Eliminationsphase zur Bewertung heranzuziehen (AGS 2021).

Die genaue Beschreibung der Methode zur Halbwertszeit-Bestimmung nach intratrachealer Gabe findet sich bei Bernstein und Riego Sintes (1999). Mindestens je sieben weibliche oder männliche Fischer-344- oder Wistar-Ratten werden durch intratracheale Instillation, die einmal täglich an vier aufeinanderfolgenden Tagen erfolgt, gut charakterisierten Fasersuspensionen ausgesetzt, die so optimiert wurden, dass sie für Ratten weitgehend einatembar sind. Die Kontrollgruppen sollten pro Beobachtungszeit je fünf Tiere enthalten und entsprechend nur Kochsalz- oder Pufferlösung verabreicht bekommen. Viermalig werden je 0,5 mg (insgesamt 2 mg) in je maximal 0,4 ml 0,9%iger Kochsalzlösung (0,3 ml Lösung in der Praxis; Schulz 2024) oder einer geeigneten Pufferlösung pro Gabe verabreicht. Die eingesetzten Fasern sollten Durchmesser von möglichst 0,8 µm haben, jedoch sollten 95 % unter 3 µm liegen. Die chemische Zusammensetzung des Fasermaterials sollte mindestens mit einer Genauigkeit von 0,5 % bekannt sein (die Inhaltsstoffe der Glaswolle sollten zu 98 % bekannt sein; EUCEB 2016; Schulz 2024). Nach Beendigung der Instillation werden Untergruppen von mindestens fünf Tieren in bestimmten Zeitabständen (zwei Tage, 14 Tage, vier Wochen und drei Monate nach der letzten Instillation als Mindestanforderung) getötet und die Lungenbelastung durch entsprechend validierte Extraktions- und Messverfahren bestimmt (mit dem REM in der Praxis; Schulz 2024). Die Messungen umfassen die Charakterisierung der Anzahl, der bivariaten Größenverteilung und der chemischen Zusammensetzung der Fasern (und Partikel) in der Lunge sowie die Halbwertszeit der Fasern, die länger als 20 μm sind. Die Halbwertszeiten der Längenfraktionen < 5 μm und 5–20 μm WHO-Fasern sollten ebenfalls aus den Daten bestimmt werden (Bernstein und Riego Sintes 1999). Zur Sicherstellung der Gleichmäßigkeit der Suspensionen werden gravimetrische Bestimmungen durchgeführt. Die Berechnung der Halbwertszeit erfolgt mit Hilfe einer nichtlinearen exponentiellen Regressionsanalyse (EUCEB 2016; Schulz 2024).

Einen Überblick über Halbwertszeiten nach intratrachealer Instillation von Glaswollen, deren Daten zur Ableitung des MAK-Wertes herangezogen wurden, geben Tabelle 3 und 4.

**Tab. 3:** Halbwertszeiten von Glaswollen in der Lunge von Ratten nach intratrachealer Gabe (Muhle et al. 1998)

**Faser**	**Dosis**	**Mittlere Halbwertszeit (95-%-KI) [Tage] für**			
		**Faseranzahl **	**WHO-Faseranzahl **	**Faseranzahl (L > 20 µm) **	**Fasermasse **
MMVF11	2 mg	217 (186–258)	199 (172–235)	107 (93–126)	126 (113–144)
B-01/09	0,35 mg	33 (28–41)	32 (26–45)	9 (7–11)	29 (24–37)
X607	2 mg	46 (41–53)	46 (40–54)	39 (33–46)	49 (40–63)

KI: Konfidenzintervall; L: Länge

**Tab. 4:** Glaswolle-Halbwertszeiten in der Lunge von Ratten nach intratrachealer Gabe

**Faser**	**Mittlere Halbwertszeit (95-%-KI) [Tage]**	**Literatur**
MMVF11	199 (172–235); WHO-Faser	Roller et al. 1996
	155 (132–178); WHO-Faser 137 (65–209); L > 20 µm; T_2_	AGS 2002
B-01/09	32 (26–45)	Roller et al. 1996
B-2L *dünner*	39 (36–42)	Bellmann et al. 1990
	37 (35–39); alle Faserlängen	Muhle et al. 1994
	18; WHO-Faser 5; L > 20 µm	AGS 2002
B-2K	38 (35–41)	Lunn et al. 2009
B-1K	107 (98–119)	Lunn et al. 2009
B-1M *dicker*	104 (96–112)	Bellmann et al. 1990
B-1L	107 (98–119)	Lunn et al. 2009
X607	45 (36–55)	AGS 2002

KI: Konfidenzintervall; L: Länge; T_2_: Halbwertszeit der langsamen Eliminationsphase bei Vorliegen einer biphasischen Eliminationskinetik

#### 3.1.2 Inhalation

Nach sechs Stunden Ganzkörper-Exposition von fünf männlichen Syrischen Hamstern gegen 30 mg **MMVF10a**/m^3^ erfolgte einen Tag später eine Lungenuntersuchung auf Faseranzahl. Im eingesetzten Aerosol befanden sich 493 WHO-Fasern/ml und 167 Fasern/ml mit einer Länge > 20 µm. In der Lunge wurden 8,4 ± 2,3 × 10^5^ WHO-Fasern und 1,6 ± 0,6 × 10^5^ Fasern mit einer Länge > 20 µm nachgewiesen (McConnell et al. 1999).

Je 56 Fischer-344-Ratten wurden gegen 0 oder 30 mg **MMVF11** oder **B-01/09**/m^3^ an fünf Tagen (6 h/d) nur über die Nase exponiert. Die Veränderungen von Faserlänge und -durchmesser wurden zu verschiedenen Zeitpunkten in der Lunge untersucht (Tabelle 5 und 6). Der Vergleich zeigt die bessere Löslichkeit der Faser B-01/09 in der Lunge (Bernstein et al. 1996). Die nach intratrachealer Gabe ermittelten Halbwertszeiten der hier eingesetzten Glaswollen betrugen für MMVF11 199 Tage und für B-01/09 32 Tage (Roller et al. 1996).

**Tab. 5:** Verhalten von MMVF11 in der Lunge von Ratten nach der Inhalation (Bernstein et al. 1996)

**Parameter**	**Zeitpunkt nach Inhalation**			
	**1 h**	**1 d**	**4 Wo**	**26 Wo**
**Fasern**				
Anzahl Fasern ausgewertet	2098	2028	2156	601
Mittlere Faseranzahl ± SD/Lunge (× 10^6^)	7,5 ± 2,2	6,0 ± 2,5	2,8 ± 1,8	0,35 ± 0,11
Mittlere WHO-Faseranzahl/Lunge (× 10^6^)	5,7	4,8	2,0	0,23
Durchmesser [µm]	0,1–3,0	0,1–2,5	0,1–2,4	0,2–3,0
Länge [µm]	1,1–65	1,2–100	1,0–40	1,3–66
Mittlerer Durchmesser ± SD [µm]	0,60 ± 0,29	0,59 ± 0,28	0,63 ± 0,33	0,67 ± 0,33
Mittlere Länge ± SD [µm]	11,4 ± 8,9	10,9 ± 8,4	8,2 ± 5,4	7,5 ± 5,1
GMD [µm] (GSD)	0,54 (1,6)	0,54 (1,6)	0,55 (1,7)	0,59 (1,6)
GML [µm] (GSD)	8,7 (2,1)	8,5 (2,0)	6,8 (1,9)	6,4 (1,7)
**Partikel**				
Anzahl Partikel ausgewertet	108	100	111	35
Mittlere Partikelanzahl/Lunge (× 10^6^)	0,42	0,35	0,14	0,02

GMD: geometrischer Mittelwert Durchmesser; GML: geometrischer Mittelwert Länge; GSD: geometrische Standardabweichung; SD: Standardabweichung

**Tab. 6:** Verhalten von Glaswolle B-01/09 in der Lunge von Ratten nach der Inhalation (Bernstein et al. 1996)

**Parameter**	**Zeitpunkt nach Inhalation**			
	**1 h**	**1 d**	**4 Wo**	**13 Wo**
**Fasern**				
Anzahl Fasern ausgewertet	2500	2769	2374	519
Mittlere Faseranzahl ± SD/Lunge (× 10^6^)	7,7 ± 1,3	7,6 ± 2,4	1,2 ± 0,8	0,35 ± 0,22
Mittlere WHO-Faseranzahl ± SD/Lunge (× 10^6^)	5,1 ± 1,3	4,6 ± 2,0	0,66 ± 0,47	0,18 ± 0,11
Durchmesser [µm]	0,1–1,7	0,1–2,5	0,1–2,2	0,1–2,2
Länge [µm]	0,7–120	0,6–45	1,0–20	1,5–25
Mittlerer Durchmesser ± SD [µm]	0,46 ± 0,22	0,46 ± 0,24	0,59 ± 0,29	0,60 ± 0,27
Mittlere Länge ± SD [µm]	7,7 ± 5,9	6,8 ± 4,7	5,7 ± 2,6	5,5 ± 2,6
GMD [µm] (GSD)	0,42 (1,6)	0,41 (1,6)	0,53 (1,6)	0,55 (1,6)
GML [µm] (GSD)	6,3 (1,9)	5,5 (1,9)	5,1 (1,6)	5,0 (1,6)
**Partikel**				
Anzahl Partikel ausgewertet	86	129	112	55
Mittlere Partikelanzahl/Lunge (× 10^6^)	0,30	0,47	0,10	0,04

GMD: geometrischer Mittelwert Durchmesser; GML: geometrischer Mittelwert Länge; GSD: geometrische Standardabweichung; SD: Standardabweichung

Die Löslichkeit/Clearance von **MMVF10** und **MMVF11** in der Lunge von Fischer-344-Ratten nach Exposition gegen 30 mg/m^3^ nur über die Nase über einen Zeitraum von fünf Tagen, sechs Stunden pro Tag, ist in Tabelle 7 nach Länge und Durchmesser getrennt dargestellt. In der Nachbeobachtungszeit wurden die langen Fasern am schnellsten abgebaut. Die eingesetzten Glaswollefasern hatten die in Tabelle 8 angegebene Zusammensetzung (die Gehalte wurden als Oxide angegeben, es liegen aber in der Glaswolle die Kationen vor) (Musselman et al. 1994). Der vermeintlich schnellere Abbau der langen Fasern ist damit zu erklären, dass die langen Fasern brechen und damit die Anzahl an kurzen Fasern erhöhen.

**Tab. 7:** Fasern in der Lunge von Ratten, 1 Tag bis 90 Tage nach Expositionsende (Faserzahl/mg Trockengewicht × 1000), Mittelwerte (± Standardabweichung) von fünf untersuchten Tieren pro Glaswolle und Zeitpunkt (Musselman et al. 1994)

**Faserdimensionen**	**Tage nach Expositionsende**			
	**1**	**5**	**31**	**90**
**MMVF10**				
Länge				
< 5 µm	8 ± 4,1	10,1 ± 2,1	3,1 ± 1,3	4,5 ± 1,4
> 5 µm	20 ± 6,4	13,9 ± 1,9	12,3 ± 2,4	6,5 ± 1,5
> 10 µm	9 ± 5,0	5,2 ± 0,8	4,8 ± 1,2	1,3 ± 0,5
> 20 µm	1,5 ± 1,7	0,6 ± 0,5	0,3 ± 0,3	0,1 ± 0,2
Durchmesser				
< 0,5 µm	4,8 ± 1,3	5,0 ± 0,6	6,0 ± 1,1	5,3 ± 1,8
> 0,5 µm	23,1 ± 1,7	19,0 ± 2,3	9,5 ± 2,6	5,7 ± 2,3
> 1,0 µm	8,0 ± 1,4	7,4 ± 0,6	4,1 ± 1,3	2,1 ± 1,2
**MMVF11**				
Länge				
< 5 µm	9,7 ± 3,0	10,4 ± 3,7	4,6 ± 1,2	3,6 ± 1,0
> 5 µm	37,6 ± 3,9	25,1 ± 3,2	15,8 ± 1,1	5,6 ± 2,5
> 10 µm	21,8 ± 2,9	13,5 ± 1,7	5,5 ± 1,1	1,7 ± 1,4
> 20 µm	4,7 ± 0,6	3,2 ± 0,7	0,4 ± 0,2	0,2 ± 0,3
Durchmesser				
< 0,5 µm	23,5 ± 2,0	19,6 ± 0,9	12,3 ± 1,0	5,7 ± 0,7
> 0,5 µm	23,1 ± 4,0	16,0 ± 2,9	8,1 ± 1,3	3,7 ± 1,3
> 1,0 µm	3,3 ± 1,5	1,9 ± 0,9	1,6 ± 0,5	0,8 ± 0,5

**Tab. 8:** Chemische Glaszusammensetzung von MMVF10 und MMVF11 (in Prozent) (Musselman et al. 1994)

**Faser**	**SiO_2_**	**B_2_O_3_**	**Na_2_O**	**Al_2_O_3_**	**FeO + Fe_2_O_3_**	**K_2_O**	**CaO**	**MgO**
MMVF10	57,5	8,8	15,0	5,1	0,1	1,1	7,5	4,1
MMVF11	63,4	4,5	15,5	3,9	0,3	1,3	7,5	2,8

In einem weiteren Versuch mit je 72 männlichen Fischer-344-Ratten wurden die Tiere fünf Tage, sechs Stunden pro Tag, gegen 30 mg/m^3^ der Fasern **MMVF10** oder **MMVF11** inhalativ exponiert. Ein Tag und 365 Tage nach Ende der Exposition wurde die Anzahl der Fasern, gruppiert nach Länge, in der Lunge bestimmt sowie die Halbwertszeiten und der prozentuale Anteil der Fasern in der Lunge berechnet (Tabelle 9). In dieser Studie zeigte MMVF11 eine kürzere Halbwertszeit und damit eine bessere Löslichkeit/Clearance in der Lunge (Hesterberg et al. 1996 b).

**Tab. 9:** Lungenbelastung und Faserclearance nach Inhalation entsprechend der Länge der Glaswollen MMVF10 und MMVF11 (Hesterberg et al. 1996 b)

**Faser**	**Faserlänge**			
	**< 5 µm**	**> 5 µm WHO-Fasern**	**> 10 µm**	**> 20 µm**
Fasern/Lunge × 10^6^ ± SD nach 1 Tag				
MMVF10	1,7 ± 0,5	2,8 ± 0,4	1,0 ± 0,2	0,14 ± 0,09
MMVF11	3,0 ± 0,5	5,6 ± 1,2	3,1 ± 0,7	0,97 ± 0,33
Fasern/Lunge × 10^6^ ± SD nach 365 Tagen				
MMVF10	0,3 ± 0,04	0,3 ± 0,1	0,1 ± 0,03	0,002 ± 0,003
MMVF11	0,3 ± 0,2	0,2 ± 0,1	0,04 ± 0,2	0,002 ± 0,003
% Fasern nach 365 Tagen Nachbeobachtungszeit				
MMVF10	18	11	8	1
MMVF11	9	4	1	< 1
Halbwertszeit [Tage]				
MMVF10	111	89	83	44
MMVF11	46	35	22	6

SD: Standardabweichung

Der Produktionsprozess der Faser **X607** ähnelt dem der keramischen Faser RCF, die allerdings mit einem sehr hohen Aluminium-Anteil eine andere chemische Zusammensetzung besitzt. X607 besitzt einen sehr hohen Siliciumdioxid-Anteil (57,9 % SiO_2_) und Calcium-Anteil (38,3 % CaO) und einen sehr geringen Aluminium-Anteil (Al_2_O_3_ 0,18 %). Die Angaben erfolgen aus analysetechnischen Gründen als Oxid, in die Glasstruktur eingebaut werden aber Aluminium- und Calcium-Ionen. Bei männlichen Fischer-344-Ratten, die zwei Jahre gegen 30 mg X607/m^3^ an sechs Stunden pro Tag und fünf Tagen in der Woche nur über die Nase exponiert waren, erhöhte sich das Lungengewicht im Vergleich zur Kontrolle erst ab der 78. Woche. Die Fasern lösten sich in „simulated extracellular fluid“ (SEF) bei pH 7,4 und 37 °C in einem Test zur Löslichkeit im In-vitro-Durchflusssystem („in vitro flow dissolution system“) mit einer Geschwindigkeitskonstante (k_dis_) von 990 ng/cm^2^/h auf. Die Auflösungskurve für X607 stimmte nicht mit der theoretischen Kurve für ein Modell mit konstanter Geschwindigkeit überein. Dies kann u. a. darauf zurückgeführt werden, dass einige Komponenten schneller aus der Faser gelöst werden als andere, was zu einer sich mit der Zeit ändernden chemischen Zusammensetzung führte. Die Fasern bestanden nach ca. 500 Stunden zu etwa 90 % aus SiO_2_ und zu ca. 10 % aus Al_2_O_3_, jedoch lag bereits nach 200 Stunden kein CaO mehr vor (siehe Abschnitt 5.2.1; Hesterberg et al. 1998).

Bei In-vitro-Messungen betrug die Lösungsgeschwindigkeitskonstante k_dis_ bei pH 7,4 von **MMVF11** (Durchmesser 0,98 µm) 100 ng/cm^2^/h und von **MMVF10** (Durchmesser 1,4 µm) 300 ng/cm^2^/h (Eastes und Hadley 1996), während sie im Vergleich < 1 ng/cm^2^/h für Amosit (Braunasbest) betrug (Hesterberg et al. 1999).

Bei **MMVF10**, **MMVF10.1** und **MMVF10a** aus verschiedenen Chargen derselben Firma betrugen die Lösungsgeschwindigkeitskonstanten k_dis_ = 300–500 ng/cm^2^/h. Für **MMVF10.1** wird k_dis_ = 300 ng/cm^2^/h angegeben (Hesterberg et al. 1999).

##### Unterschied zwischen Mensch und Ratte

Die Deposition von Fasern mit einem Durchmesser von 1 µm bezogen auf die Lungenoberfläche ist beim Menschen bei einem Atemvolumen von 20 l/min, acht Stunden pro Tag, bei Nasenatmung bis zu dreimal und bei Mundatmung bis zu sechsmal höher als bei Ratten, die sechs Stunden pro Tag exponiert sind (Nielsen und Koponen 2018). Es wird nur die Nasenatmung bei der Grenzwertableitung berücksichtigt, da Mundatmung erst ab 35 l/min einsetzt (nach MPPD-Modell; ARA 2015).

Für die normale Eliminationshalbwertszeit von GBS aus dem Alveolarbereich werden für die Ratte 60 Tage und für den Menschen 400 Tage aufgrund der Makrophagenclearance angenommen (Hartwig 2012). Von diesem Clearance-Unterschied (Faktor 6,7) ist auch bei biobeständiger Glaswolle auszugehen. Bei löslichen Substanzen ist bei der Elimination aus der Lunge nicht nur die Makrophagenclearance, sondern auch die Biolöslichkeit von Bedeutung. Daher wird für Glaswolle mit einer HWZ < 40 Tagen bei der Berechnung der humanäquivalenten Konzentration (HEC), statt eines Faktors 6,7 für biobeständige Partikel, ein Faktor von 3 für besser lösliche Partikel verwendet.

Beide von Hesterberg et al. (1993) eingesetzten Glaswollen **MMVF11** und **MMVF10** sind biobeständiger als die Glaswollen mit einer intratrachealen HWZ < 40 Tagen, was ein Kriterium für eine Einstufung einer Faser als nicht kanzerogen ist (AGS 2021).

#### 3.1.3 Dermale Aufnahme

Zur dermalen Aufnahme liegen keine Studien vor.

### 3.2 Metabolismus

#### 3.2.1 Mensch

Hierzu liegen keine bewertbaren Daten vor.

#### 3.2.2 Ratte

Das Clearance-Verhalten der in der Zwei-Jahre-Studie mit Ratten eingesetzten **MMVF10**-Glaswolle wurde mithilfe eines mathematischen Modells beschrieben. MMVF10-Fasern mit einer Länge ≤ 15 µm werden mit gleicher Geschwindigkeit von alveolären Makrophagen aufgelöst wie inerte Staubpartikel. MMVF10-Fasern mit einer Länge von 15–20 µm können brechen oder werden ebenso wie die kürzeren Fasern von alveolären Makrophagen direkt verdaut. MMVF10-Fasern mit einer Länge von > 20 µm brechen in kürzere Stücke und werden dann von den Makrophagen aufgenommen und verdaut. Die Anzahl der kurzen Fasern in der Lunge setzt sich durch die kombinierten Auswirkungen der Clearance der kurzen Fasern und der durch Brüche der langen Fasern entstandenen zusammen (Tran et al. 1996).

Der Grund für einen schnelleren Abbau von Fasern mit einer Länge > 20 µm könnte in einer größeren Zahl von Querbrüchen der Fasern liegen. Eine andere Erklärungsmöglichkeit besteht darin, dass die langen Fasern im oberen Respirationstrakt deponiert werden und infolge der im Vergleich zur alveolären Clearance schnelleren tracheobronchialen Clearance bevorzugt aus der Lunge eliminiert werden (Bellmann und Muhle 1995).

## 4 Erfahrungen beim Menschen

Die WHO-Faser hat aufgrund ihrer Dimensionen mit einem Durchmesser ≤ 3 µm, einer Länge von > 5 µm und einem Länge : Durchmesser-Verhältnis von ≥ 3:1 Eigenschaften, die in Abhängigkeit von ihrer Biobeständigkeit eine toxische und damit lungenschädliche Wirkung beim Menschen verursachen können (Hartwig und MAK Commission 2018).

### 4.1 Einmalige Exposition

Hierzu liegen keine Daten vor.

### 4.2 Wiederholte Exposition

Die alveolengängigen Faserkonzentrationen im Atembereich wurden bei 52 bis 70 Beschäftigten in einem Glasfaser-produzierenden Betrieb mit 1,3–5,5 Fasern/ml angegeben. Die Beschäftigten waren 12 bis 24 Jahre im Betrieb tätig. Im Vergleich mit nicht exponierten angepassten Kontrollpersonen zeigten sich keine Unterschiede in den Lungenfunktionsmessungen, bei der Auswertung von Röntgenaufnahmen und bei den mithilfe eines Fragebogens („Medical Research Council Short Questionnaire on Respiratory Symptoms (1960)“) abgefragten Symptomen am Atemtrakt (Hill et al. 1973). Die Methoden sind nicht ausführlich beschrieben und die Bewertungskriterien insbesondere der Röntgenaufnahmen entsprechen nicht dem heutigen Standard. Entzündliche Effekte wurden in der Studie nicht untersucht. Die Studie wird daher nicht zur Bewertung herangezogen.

Die Effekte verschieden hoher Faserkonzentrationen auf das respiratorische System wurden bei 1028 Beschäftigten aus sieben Betrieben, in denen ausschließlich Glaswolle produziert wurde, untersucht. Es wurden Lungenfunktionsmessungen, Röntgenaufnahmen und eine Befragung mittels Fragebogen (angepasste Version des „American Thoracic Society Division of Lung Diseases Questionaire“) durchgeführt. In der Gruppe der Nichtraucher wiesen die Lungenfunktionsmessungen keine veränderten Werte auf. Nur in der Gruppe der rauchenden Beschäftigten zeigten Röntgenaufnahmen verstärkt Trübungen in Abhängigkeit von der Expositionskonzentration an Fasern. Diese Gruppe berichtete auch über mehr Symptome im respiratorischen System (Weill et al. 1983). Obwohl die hier durchgeführten Lungenfunktionsmessungen keine Einschränkungen bei Nichtrauchern ergaben, kann die Studie nicht zur Bewertung herangezogen werden, da das Auftreten von frühen Entzündungsmarkern mit dieser Methode nicht beobachtet werden kann.

Die vorliegenden epidemiologischen Studien sind nicht ausreichend, um die kanzerogene Wirkung von Glaswolle (HWZ < 40 d) zu bewerten (NTP 2021).

### 4.3 Wirkung auf Haut und Schleimhäute

In einem Betrieb, der Glaswolle produzierte, gaben Beschäftigte in einem Fragebogen an, dass sie Hautausschläge (Glasfaserdermatitis) in den ersten Monaten ihrer Beschäftigung hatten; diese wurden häufiger angegeben als in der Kontrollgruppe. Die Beschäftigten waren zum Untersuchungszeitpunkt gegen 1,3–5,5 alveolengängige Fasern/ml und 0,6–11,6 mg Gesamtstaub/m^3^ exponiert und im Mittel 19,85 Jahre beschäftigt (Abschnitt 4.2; Hill et al. 1973). Beim Umgang mit Glasfasern kann es durch das Eindringen in die Haut und der damit verbundenen mechanischen Reizung zur sogenannten Glasfaserdermatitis kommen. Die am häufigsten betroffenen Stellen sind Hände, Gesicht, Nacken, Unterarme und Hautfalten. Insbesondere Fasern mit einem Durchmesser von mehr als 4,5 µm können Hautreizungen verursachen, wobei das Ausmaß direkt proportional zum Durchmesser und umgekehrt proportional zur Länge der Glasfasern ist. Als Symptome treten starker Juckreiz sowie unter anderem Rötungen, Bläschen, Schuppung auf; seltener wird über Brennen, Rissbildung, Ulzeration und postinflammatorische Hyperpigmentierung berichtet. Die Glasfaserdermatitis ist in der Regel akut. Eine chronische Erkrankung ist selten, da eine längere Exposition zu einer Toleranz der Betroffenen führen kann (Camacho et al. 2019). Die Toleranz scheint insbesondere das subjektive Symptom des Juckreizes zu betreffen (Sertoli et al. 2000).

### 4.4 Allergene Wirkung

#### 4.4.1 Hautsensibilisierende Wirkung 

Hautkontakt gegenüber Glaswolle könnte durch direkten Umgang und durch Kontakt mit kontaminierten Bereichen, Kleidung und persönlicher Schutzausrüstung, aber auch durch Fasern in der Luft erfolgen.

Analysen der chemischen Zusammensetzung von Glaswollen (McConnell et al. 1999) weisen auf das Vorhandensein verschiedener Metalle, die potenziell sensibilisierend sein können, hin. Die Freisetzung von Metallionen nach Hautkontakt kann nicht vollständig ausgeschlossen werden. Daten zur sensibilisierenden Wirkung von Glaswolle (HWZ < 40 d) liegen jedoch weder für den Menschen noch aus tierexperimentellen Untersuchungen vor.

Es liegen einige wenige Fälle allergischer Kontaktdermatitis im Zusammenhang mit beschichteter Glaswolle vor. Hierbei wurden die Monomere von Formaldehyd- oder Epoxid-Harzen oder Molybdän, welche zur Beschichtung von Glaswolle eingesetzt wurden, als allergieauslösende Komponente identifiziert (Übersicht s. Sertoli et al. 1992; aktuellerer Fall mit Molybdän: Navarro-Triviño et al. 2021; Nogueira et al. 2011). Beschichtete Glaswolle ist jedoch nicht Gegenstand dieser Begründung.

#### 4.4.2 Atemwegssensibilisierende Wirkung

Hierzu liegen keine Daten vor.

### 4.5 Reproduktionstoxizität

Hierzu liegen keine Daten vor.

### 4.6 Genotoxizität

Hierzu liegen keine Daten vor.

### 4.7 Kanzerogenität

Hierzu liegen keine Daten vor.

## 5 Tierexperimentelle Befunde und In-vitro-Untersuchungen

### 5.1 Akute Toxizität

Hierzu liegen keine Daten vor.

### 5.2 Subakute, subchronische und chronische Toxizität

#### 5.2.1 Inhalative Aufnahme

Zu Glaswolle (HWZ < 40 d) liegen keine Daten vor. Es werden daher Glaswollen betrachtet, deren Biobeständigkeit höher, aber als gering im Vergleich mit Asbestfasern eingeschätzt wird.

Die Studien sind im Detail in Tabelle 15 dargestellt.

##### 5.2.1.1 Ratte

Je zwölf männliche Fischer-344-Ratten wurden vier oder zwölf Wochen nur über die Nase vier Stunden am Tag und fünf Tage pro Woche gegen 0 mg oder 44 mg **MMVF10a**/m^3^ (761 Fasern/ml, davon 610 WHO-Fasern/ml) exponiert. Weitere zwölf Tiere wurden nach zwölfwöchiger Exposition zwölf Wochen nachbeobachtet. Der geometrische Mittelwert der Länge (GML) der Fasern betrug 12,5 µm (geometrische Standardabweichung (GSD) 2,5) und der geometrische Mittelwert des Durchmessers (GMD) 0,9 µm (GSD 0,52). Im Aerosol betrug der Partikelanteil ca. 13 %. In Tabelle 10 sind die Faserbelastungen in Lunge und Pleura dargestellt. Die Fasern in der Lunge waren kürzer und dünner als im eingesetzten Aerosol. In der Pleura fanden sich deutlich weniger Fasern als in der Lunge. In der bronchoalveolären Lavageflüssigkeit (BALF) traten bereits nach vier Wochen signifikant erhöht Neutrophile und Lymphozyten auf, dies verstärkte sich nach zwölf Wochen. Es wurden zudem signifikante Anstiege der Laktat-Dehydrogenase (LDH)-, der N-Acetyl-β-D-Glucosaminidase (NAG)- und der Alkalischen-Phosphatase-Aktivitäten sowie des Gesamtproteins beobachtet. Die Lavage des Pleuralspaltes ergab eine signifikant erhöhte Anzahl an Makrophagen bereits nach vierwöchiger Exposition und einen Anstieg der LDH-Aktivität nach zwölf Wochen. In Tabelle 15 sind die genauen Daten dargestellt. Die Autoren interpretieren die Ergebnisse in der Pleura als minimale Entzündung (Bermudez et al. 2003).

**Tab. 10:** Faserbelastung nach MMVF10a-Exposition in Lunge und Pleura bei Ratte und Hamster (Bermudez et al. 2003)

**Woche**	**Länge [µm]**	**Faserzahl pro cm^2^ Lungenoberfläche^a)^ (× 10^2^)**		**Faserzahl pro cm^2^ Pleuraoberfläche^a)^ (× 10^2^)**	
		**Hamster**	**Ratte**	**Hamster**	**Ratte**
0	1 < L ≤ 3				
	3 < L ≤ 5				
	5 < L ≤ 8		0,2	0,0	0,0
	> 8	0,4	0,2	0,0	0,0
4	1 < L ≤ 3	4,5 ± 1,8	5,9 ± 2,0		0,0
	3 < L ≤ 5	8,0 ± 1,9	13,0 ± 2,6	0,0	0,0
	5 < L ≤ 8	8,9 ± 2,3	14,3 ± 3,2	0,1 ± 0,0	0,1 ± 0,1
	> 8	15,6 ± 4,9	33,0 ± 6,7	0,4 ± 0,1	0,2 ± 0,0
12	1 < L ≤ 3	2,4 ± 0,3	8,5 ± 1,9	0,1 ± 0,0	0,0
	3 < L ≤ 5	7,4 ± 0,9	25,4 ± 4,6	0,2 ± 0,1	0,1 ± 0,0
	5 < L ≤ 8	7,3 ± 0,7	25,7 ± 2,4	0,4 ± 0,2	0,2 ± 0,0
	> 8	11,8 ± 1,7	44,0 ± 7,7	0,9 ± 0,2	0,3 ± 0,1
12/12	1 < L ≤ 3	0,7 ± 0,4	7,0 ± 1,8	0,0	0,0
	3 < L ≤ 5	0,9 ± 0,2	14,6 ± 2,4	0,0	0,1 ± 0,0
	5 < L ≤ 8	0,7 ± 0,2	16,3 ± 2,1	0,1 ± 0,0	0,3 ± 0,1
	> 8	0,3 ± 0,1	20,2 ± 2,0	0,5 ± 0,1	0,3 ± 0,1

^a)^ mittlere Anzahl ± Standardfehler

Je 31 oder 35 männliche Fischer-344/N-Ratten wurden bis zu 13 Wochen, an fünf Tagen pro Woche und sechs Stunden täglich, gegen 0; 3,2; 16,5; 30,5; 44,5 oder 62,2 mg **MMVF10**/m^3^ nur über die Nase exponiert. Diese gravimetrisch ermittelten Konzentrationen entsprachen Faserzahlen von 0, 36, 206, 316, 550 oder 714 Fasern/ml, das entspricht 12 Fasern/ml per mg/m^3^. Je sechs Tiere wurden sieben oder zehn Wochen nachbeobachtet. Im Aerosol waren auch sehr lange oder sehr dicke Fasern enthalten, die nicht eingeatmet werden können und sich daher auch nicht in der Lunge wiederfinden lassen. Die aus der Lunge gewonnenen Fasern wiesen keine signifikanten konzentrations- oder zeitabhängigen Dimensionsunterschiede auf. Bei 30,5 mg MMVF10/m^3^ war der GMD der Fasern im Aerosol und nach 13 Wochen in der Lunge 0,87 bzw. 0,13 µm, der GML 13,1 bzw. 5,5 µm. Die Anzahl der Fasern in der Lunge erhöhte sich mit zunehmender Konzentration und längerer Expositionszeit und reduzierte sich wieder in der Nachbeobachtungszeit. Auch bei der niedrigsten Konzentration von 3,2 mg/m^3^ nahm die Anzahl der WHO-Fasern deutlich zu und ging auch nach zehnwöchiger Nachbeobachtungszeit nicht vollständig zurück, siehe Tabelle 11. Die Clearance-Halbwertszeiten wurden mithilfe radioaktiv markierter ^85^Sr-Mikropartikel bestimmt und reichten von 34 ± 1 Tagen in der Kontrollgruppe bis zu 51 ± 3 Tagen in der Expositionsgruppe mit 714 Fasern/ml (62,2 mg/m^3^). In der BALF wurde nach 13-wöchiger Exposition ein Anstieg der LDH- und NAG-Aktivitäten sowie der Gesamtproteinmenge beobachtet, jedoch ohne Dosisabhängigkeit (k. w. A.). Der Gehalt an Lymphozyten und Neutrophilen nahm dagegen dosisabhängig mit statistischer Signifikanz ab 16,5 mg/m^3^ zu. Die mittlere Anzahl der Neutrophilen betrug 0,07 ± 0,05 × 10^6^/Lunge für die Kontrollen, 0,54 ± 0,27 × 10^6^/Lunge für die 30,5-mg/m^3^-Gruppe und 0,78 ± 0,08 × 10^6^/Lunge für die 62,2-mg/m^3^-Gruppe. Die Anzahl an Makrophagen stieg nicht an. Die Proliferation in der Lunge wurde mithilfe des „proliferating cell nuclear antigen“ (PCNA) bestimmt. PCNA-immunreaktive Zellkerne waren am häufigsten in den Epithelzellen der terminalen Bronchiolen und Alveolargänge zu finden, aber auch in Alveolarmakrophagen, Endothel- und glatten Muskelgefäßzellen sowie interstitiellen Zellen. Proliferierende Zellen waren an den Verzweigungen der Bronchiolen und in den Alveolargängen am zahlreichsten, das heißt in Bereichen, in denen die faserinduzierten entzündlichen Veränderungen am deutlichsten waren. Dieser Effekt war ab 16,5 mg/m^3^ erhöht. Weitere Organgewichte waren unverändert. Nach sieben Wochen wurden ab 30,5 mg/m^3^ ein unnormaler Thoraxkollaps bei Öffnung und ab 62,2 mg/m^3^ Foci (2/6) beobachtet. Fibrosen wurden bei einzelnen Ratten in den Nachbeobachtungsgruppen nachgewiesen. Die genauen Daten sind in Tabelle 15 dargestellt (Hesterberg et al. 1996 a).

Nach 13-wöchiger Exposition von je drei bis sechs Fischer-344-Ratten gegen 30 mg Fasern/m^3^ nur über die Nase und 78 Wochen Erholungszeit sank die Faseranzahl/mg Lunge um 95 % bei **MMVF11** und um 88 % bei **MMVF10** (Hesterberg et al. 1994).

**Tab. 11:** Lungenbelastung mit WHO-Fasern der Glaswolle MMVF10 bei Ratten (Hesterberg et al. 1996 a)

**Konzentration im Aerosol**		**Faserzahl in der Lunge [WHO-Fasern × 10^6^/Lunge]**		
**mg/m^3^**	**WHO-Fasern/ml**	**7 Wochen**	**13 Wochen**	**13 Wochen und 10 Wochen Nachbeobachtung**
3	36	n. d.	2,33	1,07
16	206	n. d.	11,60	5,65
30	316	9,47	19,60	6,80
45	553	18,00	31,50	15,5
60	714	22,50	35,10	16,10

n. d.: nicht durchgeführt

Je 140 männliche Fischer-344/N-Ratten pro Faser wurden bis zu 24 Monate, an fünf Tagen pro Woche und sechs Stunden täglich, gegen 0; 3,1; 17,1 oder 27,8 mg **MMVF10**/m^3^ oder 0; 4,8; 15,8 oder 28,3 mg **MMVF11**/m^3^ nur über die Nase exponiert. Diese gravimetrisch bestimmten Konzentrationen entsprachen durchschnittlichen WHO-Faserzahlen (± SD) von 0; 29 ± 7,5; 145 ± 35 bzw. 232 ± 56 Fasern/ml für MMVF10 und 41 ± 29, 153 ± 69 bzw. 246 ± 76 Fasern/ml für MMVF11. Bei je drei oder sechs Tieren wurden nach 3, 6, 12, 18 oder 24 Monaten die histopathologischen Veränderungen der Lunge und die Faserbelastung untersucht. Weiterhin wurde bei je sechs Tieren nach 3, 6, 12 oder 18 Monaten die Exposition gestoppt. Diese Tiere wurden erst nach insgesamt 24 Monaten und damit nach unterschiedlich langen Nachbeobachtungszeiten untersucht. Alle Tiere, die mindestens ein Jahr exponiert waren, wurden auf neoplastische Veränderungen geprüft. Um das Auftreten von Mesotheliomen erfassen zu können, wurden Tiere, die 24 Monate exponiert waren, erst am Ende ihrer Lebenszeit untersucht. Die Anzahl an MMVF10- und MMVF11-Glaswollefasern in der Lunge erhöhte sich mit zunehmender Konzentration und längerer Expositionszeit. Auch bei der niedrigsten Konzentration von 3,1 mg/m^3^ erhöhte sich die Anzahl der gesamten Fasern und der WHO-Fasern deutlich (Tabelle 12). Mit zunehmender Dauer der Erholungsphase nahm die Faseranzahl in der Lunge bei allen Fasertypen ab. Durchmesser und Länge der in der Lunge deponierten Fasern waren kleiner verglichen mit den Fasern im Aerosol. Die GML und GMD der in den Lungen gefundenen Fasern zeigten kaum bzw. keine Veränderungen bei längerer Expositionsdauer, weder während der Expositionsphase noch während der Erholungsphase (Tabelle 13). Nach dreimonatiger Exposition gegen MMVF10 und MMVF11 wurde bereits ein zeitabhängiger Anstieg an Makrophagen in den terminalen Bronchiolen und proximalen Alveolen beobachtet. Nach zweijähriger Exposition wurden die pulmonalen Veränderungen in der Gruppe mit niedriger Konzentration für beide Fasertypen mit einem Schweregrad von 2,2 bzw. 2,5 nach Wagner und in der Gruppe mit mittlerer und hoher Dosis mit 2,7–3,0 bzw. 2,5–2,7 eingestuft. Da der Durchschnitt höher als 2 (= nur Makrophagenansammlung) ist, bedeutet dies, dass einige Tiere Schweregrad 3 nach Wagner hatten (= Bronchiolisation oder Entzündungen). Fibrosen traten nicht auf. Fasern konnten in vielen Makrophagen und im Interstitium nachgewiesen werden (siehe Tabelle 15). Weder in der Nasenhöhle, dem Kehlkopf, der Luftröhre, den oberen Atemwegen noch einem anderen Organ/Gewebe wurden behandlungsbedingte Läsionen gefunden. Kurze Fasern und Faserfragmente wurden innerhalb großer Makrophagen in den mediastinalen Lymphknoten beobachtet (siehe Abschnitt 5.7.2, Hesterberg et al. 1993). Das Lungengewicht der Tiere, die gegen 27,8 mg MMVF10/m^3^ exponiert waren, stieg ab der 78. Woche stark an und betrug nach 104 Wochen 23 % mehr als bei den Kontrolltieren. Auch die Faserzahl in der Lunge stieg ab der 78. Woche bei den beiden hohen Konzentrationen stark an. Dieser Anstieg wurde durch eine reduzierte Clearance verursacht, da in der 26-wöchigen Nachbeobachtungszeit die WHO-Faserzahl fast nicht abnahm (Hesterberg et al. 1996 a). In dieser Studie liegt die LOAEC für Glaswolle MMVF10 bei 3,1 mg/m^3^, die für Glaswolle MMVF11 bei 4,8 mg/m^3^. Die Daten zeigen, dass die Clearance-Kapazität des respiratorischen Systems für diese Konzentrationen nicht ausreichte, sodass auch bei der niedrigsten Konzentration von 3,1 bzw. 4,8 mg Fasern/m^3^ eine Anreicherung in der Lunge stattfand. Ein Abbau dieser Fasern durch Brüche und Auflösen der Fasern fand in der Lunge nicht statt, was sich an den unveränderten Längen und Durchmessern der Fasern nach 24-monatiger Exposition ablesen lässt.

**Tab. 12:** Belastung mit Fasern in den Lungen von Ratten bei verschiedenen Expositionskonzentrationen und -zeiten (Hesterberg et al. 1993)

**Exposition [Mo]**	**Faserzahl × 10^5^/mg Lunge (Trockengewicht)**			
	**MMVF10**		**MMVF11**	
	**Fasern gesamt**	**WHO-Fasern**	**Fasern gesamt**	**WHO-Fasern**
30 mg/m^3^				
3	1,40 ± 0,34	0,66 ± 0,18	3,72 ± 0,57	2,48 ± 0,28
6	2,31 ± 0,96	1,10 ± 0,48	3,65 ± 0,35	2,51 ± 0,25
12	2,39 ± 0,36	1,71 ± 0,27	3,47 ± 0,61	2,77 ± 0,50
18	2,50 ± 0,51	1,55 ± 0,34	3,88 ± 0,20	2,24 ± 0,18
24	4,16 ± 0,89	2,88 ± 0,56	6,42 ± 3,10	5,03 ± 2,91
16 mg/m^3^				
3	0,55 ± 0,06	0,27 ± 0,05	2,09 ± 0,07	1,58 ± 0,16
6	0,96 ± 0,35	0,50 ± 0,28	2,78 ± 0,41	1,6 ± 0,14
12	1,15 ± 0,23	0,87 ± 0,21	2,35 ± 0,69	1,83 ± 0,62
18	1,42 ± 0,31	0,95 ± 0,21	2,69 ± 0,50	1,85 ± 0,37
24	2,69 ± 0,48	1,85 ± 0,53	3,46 ± 0,86	2,35 ± 0,63
3 mg/m^3^				
3	0,16 ± 0,05	0,08 ± 0,04	0,35 ± 0,09	0,26 ± 0,03
6	0,21 ± 0,03	0,10 ± 0,02	0,42 ± 0,08	0,12 ± 0,04
12	0,27 ± 0,03	0,19 ± 0,03	0,4 ± 0,14	0,29 ± 0,11
18	0,33 ± 0,06	0,22 ± 0,04	0,46 ± 0,06	0,3 ± 0,05
24	0,37 ± 0,13	0,24 ± 0,08	0,62 ± 0,13	0,48 ± 0,11

Mo: Monate

**Tab. 13:** GMD und GML der eingesetzten Aerosole und der Fasern in den Lungen von Ratten nach Exposition gegen 30 mg/m^3^ (Hesterberg et al. 1993)

**Dimension**	**Aerosol**		**Lunge**	
	**MMVF10**	**MMVF11**	**MMVF10 **	**MMVF11**
			**3 Mo Exposition:**	
GMD [µm] (GSD)	1,26 (1,81)	0,69 (2,10)	0,62 (1,78)	0,45 (1,90)
			**24 Mo Exposition:**	
			0,42 (2,07)	0,47 (1,62)
			**3 Mo Exposition und 21 Mo Nachbeobachtung**	
			0,52 (1,58)	0,34 (1,92)
			**3 Mo Exposition:**	
GML [µm] (GSD)	13,1 (2,0)	13,7 (2,2)	5,27 (1,75)	6,89 (1,87)
			**24 Mo Exposition:**	
			6,97 (1,78)	7,34 (1,64)
			**3 Mo Exposition und 21 Mo Nachbeobachtung**	
			6,26 (1,45)	6,66 (1,46)

GMD: geometrischer Mittelwert Durchmesser; GML: geometrischer Mittelwert Länge; GSD: geometrische Standardabweichung; Mo: Monate

Für einen Vergleich der Wirkstärke verschiedener Fasern wurde eine weitere Studie, in der Ratten gegen 10 mg **Chrysotil-Asbest**/m^3^ oder 30 mg **RCF1-Faser**/m^3^ exponiert waren, herangezogen. Die Messung der Zusammensetzung der Fasertypen ergab geringe Mengen an Aluminiumoxid mit 5,17 % bei **MMVF10** und 3,79 % bei **MMVF11**, jedoch 48 % bei RCF1. Die Fasern wurden für die Aerosolierung aufbereitet und unterschieden sich danach jedoch nur gering von der Stammfaser. Die RCF-Faser führte bei den exponierten Tieren nach sechs Monaten zu Fibrosen in der Lunge (Wagner-Einstufung 4,0 = geringfügige Fibrosen) und Chrysotil-Asbest-Fasern bereits nach dreimonatiger Exposition. Da die Länge und der Durchmesser der Chrysotil-Asbestfaser sich sehr von den anderen Fasern unterscheiden, ist eine Vergleichbarkeit der toxischen Effekte jedoch nicht zweckmäßig (Hesterberg et al. 1993). Die Effekte von MMVF10 und MMFV11 sind in der Lunge damit deutlich schwächer ausgeprägt als die Effekte der RCF-Faser.

Je 24 männliche und weibliche Han-Wistar-Ratten wurden 12 oder 24 Monate gegen 0 oder 5,2 mg bzw. 4,96 mg **SG Glaswolle**/m^3^ in alveolengängiger Form (21,9 bzw. 21,2 mg/m^3^ als einatembarer Staub („total dust“)) ganzkörperexponiert. Ein Teil der Tiere wurde 7, 12 oder 16 Monate nachbeobachtet. Die alveolengängige Fraktion enthielt 70 Fasern/ml und 48 Fasern/ml mit einer Länge > 5 µm. In der Expositionskammer lag der Anteil der Fasern mit einem Durchmesser von < 2 µm bei 84 % und Fasern mit einer Länge < 5 µm machten 10,8 % an der Gesamtzahl aus. Die Fasermenge in der Lunge stieg von 2,12 mg/g Lungengewebe nach zwölf Monaten Exposition auf 3,19 mg/g nach 24 Monaten Exposition, in den Lymphknoten von 0,23 mg/g Gewebe auf 0,96 mg/g und in der Trachea von 0,15 mg/g Gewebe auf 1,13 mg/g. Nach zwölf Monaten wurde Makrophagenakkumulation in der Lunge beobachtet. Nach 24 Monaten traten Entzündungen in der Nase auf. Die genauen Daten sind in Tabelle 15 aufgeführt. Trachea, Larynx sowie weitere untersuchte Organe waren ohne Auffälligkeiten. Die Zusammensetzung dieser Glaswolle ist in Tabelle 14 angegeben. Die Gehalte wurden als Oxide angegeben, es liegen aber in der Glaswolle die Kationen vor (Le Bouffant et al. 1987). Die Halbwertszeit nach intratrachealer Instillation wurde für diese Faser nicht bestimmt.

**Tab. 14:** Chemische Glaszusammensetzung der SG-Glaswolle (in Prozent) (Le Bouffant et al. 1987)

**Faser**	**SiO_2_**	**B_2_O_3_**	**Na_2_O**	**Al_2_O_3_**	**Fe_2_O_3_**	**K_2_O**	**CaO**	**MgO**	**TiO_2_**
SG Glaswolle	68,30	4,18	13,30	3,27	0,59	1,25	8,50	0,21	0,07

In einer Zwei-Jahre-Inhalationsstudie wurde die Faser **X607** eingesetzt. Diese Faser wurde wie keramische Fasern (RCF) produziert, enthielt aber einen sehr hohen Silicium-Anteil und nur 0,2 % Aluminium. Je zehn oder mehr (k. w. A.) männliche Fischer-344N-Ratten wurden zwei Jahre lang, an sechs Stunden pro Tag, fünf Tage pro Woche, nur über die Nase gegen 0 oder 30 mg X607/m^3^ exponiert. Das Lungengewicht bei den exponierten Tieren lag ab der 78. Woche gering, jedoch nicht statistisch signifikant, über dem der Kontrollen. Daraus schließen die Autoren auf eine hohe Löslichkeit der Glaswolle in der Lunge. Bereits nach 13 Wochen wurde Makrophagen-Aggregation in der Lunge beobachtet. Nach 78 Wochen kam es zu alveolärer Bronchiolisation und Mikrogranulomen. Die Ergebnisse sind in Tabelle 15 dargestellt. Ab der 13. Woche stiegen die Effekte nach der Wagner-Einstufung von 2,0 auf 3,0 nach 52 Wochen (Hesterberg et al. 1998). Die Halbwertszeit nach intratrachealer Gabe der Faser X607 an Ratten betrug 46 Tage (AGS 2002).

Eine Zwei-Jahre-Studie mit Exposition von sechs Stunden täglich und an fünf Tagen pro Woche gegen 0; 0,3 oder 3 mg/m^3^ der Glasfaser **0,45-µm MD (**Manville Code 100 Glasfaser) wurde mit weiblichen Osborne-Mendel-Ratten durchgeführt. Im Vergleich zur Kontrolle traten keine erhöhten bronchoalveolären Metaplasien, Fibrosen oder Tumoren in der Lunge auf. Der GMD betrug 0,4 µm und die GML 4,7 µm. Die Konzentration von 3 mg/m^3^ entsprach ca. 3000 Fasern/ml und 0,3 mg/m^3^ ca. 300 Fasern/ml (Smith et al. 1987). Im Vergleich mit den Studien mit anderen Fasern ist hier die Faseranzahl pro ml ca. 10-fach so hoch. Dies kann auf die sehr kurzen Fasern zurückgeführt werden. Der Aluminiumgehalt der Faser wurde nicht ermittelt.

**Tab. 15:** Toxizität der Glaswollefasern MMVF10, MMVF11, X607 und SG-Glaswolle nach wiederholter inhalativer Exposition

**Spezies,** **Stamm,** **Anzahl pro Gruppe**	**Exposition**	**Befunde^a)^**	**Literatur**
Ratte, F344N, 6/12 ♂	4 Wo, 0, 44 mg **MMVF10a**/m^3^ (0, 610 WHO-Fasern/ml), 4 h/d, 5 d/Wo, nur über die Nase	**44 mg/m^3^**: Pleura diaphragmatica: mesotheliale Zellreplikation ↑, BALF: Neutrophile ↑, Lymphozyten ↑, LDH ↑, NAG ↑, AP ↑, Gesamtprotein ↑, PLF: Makrophagen ↑	Bermudez et al. 2003
Ratte, F344N, 6 ♂	7 Wo, 0; 3,2; 16,5; 30,5; 44,5; 62,2 mg **MMVF10**/m^3^ (0, 36, 206, 316, 550, 714 Fasern/ml), 6 h/d, 5 d/Wo, Nachbeobachtung: 10 Wo, nur über die Nase	**ab 3,2 mg/m^3^**: BALF: LDH ↑, NAG ↑, Neutrophile ↑ n. s.; **ab 16,5 mg/m^3^**: BALF: Lymphozyten ↑; **30,5 mg/m^3^**: terminale Bronchiolen: alveoläre Makrophagen ↑ (gering); **44,5 mg/m^3^**: terminale Bronchiolen: alveoläre Makrophagen ↑ (mäßig); **62,2 mg/m^3^**: Lunge: unnormaler Thoraxkollaps bei Öffnung, Foci (2/6), terminale Bronchiolen: alveoläre Makrophagen ↑ (stark)	Hesterberg et al. 1996 a
Ratte, F344N, 6/12 ♂	12 Wo, 0, 44 mg **MMVF10a**/m^3^ (0, 610 WHO-Fasern/ml), 4 h/d, 5 d/Wo, Nachbeobachtung: 12 Wo, nur über die Nase	**44 mg/m^3^**: BALF: Neutrophile ↑, Lymphozyten ↑, LDH ↑, AP ↑, Gesamtprotein ↑, PLF: Makrophagen ↑; **12 Wo Nachbeobachtung**: BALF: Neutrophile ↑, LDH ↑, AP ↑, Gesamtprotein ↑, PLF: Makrophagen ↑	Bermudez et al. 2003
Ratte, F344N, 10 ♂ nach 13 Wo, 6 ♂ nach 19 Wo, 6 ♂ nach 23 Wo	13 Wo, 0; 3,2; 16,5; 30,5; 44,5; 62,2 mg **MMVF10**/m^3^ (0, 36, 206, 316, 550, 714 Fasern/ml), 6 h/d, 5 d/Wo, Nachbeobachtung: 7, 10 Wo, nur über die Nase	**ab 3,2 mg/m^3^**: Lunge: alveoläre Makrophagen ↑, Mikrogranulome (1/5), Zellproliferation ↑ n. s.; **ab 16,5 mg/m^3^**: Lunge: alveoläre Makrophagen ↑, Mikrogranulome (4/6), Zellproliferation (am stärksten an der bronchiolären-alveolären Verzweigung) ↑, BALF: Neutrophile ↑, Lymphozyten ↑, LDH ↑, Gesamtprotein ↑; **ab 30,5 mg/m^3^**: Lunge: Mikrogranulome (6/6); **ab 44,5 mg/m^3^**: Lunge: ungewöhnliche Makrophagenverklumpungen; **7 Wo Nachbeobachtung: ** **ab 30,5 mg/m^3^**: Lunge: Mikrogranulome; **ab 44,5 mg/m^3^**: Lunge: Metaplasien (Bronchiolisation) (2/6), interstitielle Fibrose (1/6); **10 Wo Nachbeobachtung:** **ab 16,5 mg/m^3^**: Lunge: Metaplasien (Bronchiolisation) (1/6), Fibrose (1/6, minimal); **ab 30,5 mg/m^3^**: Lunge: Mikrogranulome (4/6); **ab 44,5 mg/m^3^**: Lunge: ungewöhnliche Makrophagenverklumpungen	Hesterberg et al. 1996 a
Ratte, F344N, 3 ♂	13 Wo, 0; 3,1; 17,1; 27,8 mg **MMVF10**/m^3^ (0; 29 ± 7,5; 145 ± 35; 232 ± 56 Fasern/ml), 6 h/d, 5 d/Wo, nur über die Nase	**ab 3,1 mg/m^3^**: Lunge: Makrophagen ↑ (Schweregrad^b)^ 2,0 von 8); **27,8 mg/m^3^**: Lunge: Mikrogranulome (Schweregrad^b)^ 2,0 von 8)	Hesterberg et al. 1993
Ratte, F344N, 3 ♂	13 Wo, 0; 4,8; 15,8; 28,3 mg **MMVF11**/m^3^ (0, 41 ± 29, 153 ± 69, 246 ± 76 Fasern/ml), 6 h/d, 5 d/Wo, nur über die Nase	**ab 4,8 mg/m^3^**: Lunge: Makrophagen ↑ (Schweregrad^b)^ 2,0 von 8); **28,3 mg/m^3^**: Lunge: Mikrogranulome, Foci alveolärer Bronchiolisation (Schweregrad^b)^ 3,0 von 8)	
Ratte, Han-Wistar, 24 ♂, 24 ♀	12 Mo, 5,2 mg **SG Glaswolle**/m^3^, 5 h/d, 5 d/Wo, Nachbeobachtung: 7, 12, 16 Mo, Ganzkörper	**5,2 mg/m^3^**: Lunge: Makrophagen ↑, Hyperplasie, Nase: Mukushypersekretion, Satelliten-Lymphknoten: Makrophagen ↑; **7 Mo Nachbeobachtung**: Lunge: Koniophagenakkumulation (minimal), Lymphknoten: Makrophagenakkumulation; **12 Mo Nachbeobachtung**: Nase: Entzündung; **16 Mo Nachbeobachtung**: Nase: Hyperplasie	Le Bouffant et al. 1987
Ratte, Han-Wistar, 24 ♂, 24 ♀	24 Mo, 4,96 mg **SG Glaswolle**/m^3^, 5 h/d, 5 d/Wo, Nachbeobachtung: 12 Mo, Ganzkörper	**4,96 mg/m^3^**: Lunge: Makrophagen ↑, Septumfibrosen, Nase: Entzündung, Niere: progressive chronische Läsionen; **12 Mo Nachbeobachtung**: Lunge: Makrophagen-Alveolitis (minimal), Septumfibrosen (gering)	Le Bouffant et al. 1987
Ratte, F344N, > 10 ♂	24 Mo, 0, 30 mg **X607**/m^3^, 6 h/d, 5 d/Wo, Nachbeobachtung: 23 Wo, nur über die Nase	**30 mg/m^3^**: Makrophagenaggregation ↑ (ab 13. Wo), alveoläre Bronchiolisation ↑ (ab 78. Wo), Mikrogranulome ↑ (ab 78. Wo) (Schweregrad^b)^ 3 von 8); **23 Wo Nachbeobachtung**: Makrophagenaggregation ↑, Mikrogranulome ↑, bronchoalveoläre Hyperplasie (1/121)	Hesterberg et al. 1998
Ratte, F344N, 6 ♂	24 Mo, 0; 3,1; 17,1; 27,8 mg **MMVF10**/m^3^ (0; 29 ± 7,5; 145 ± 35; 232 ± 56 Fasern/ml), 6 h/d, 5 d/Wo, nur über die Nase	**ab 3,1 mg/m^3^**: **LOAEC** Lunge: Makrophagen ↑ (Schweregrad^b)^ 2,5 von 8), rel. Gewicht ↑, Lungenclearance eingeschränkt (ab 78. Wo); **ab 17,1 mg/m^3^**: Lunge: Makrophagen ↑, Mikrogranulome, Foci alveolärer Bronchiolisation (Schweregrad^b)^ 3 von 8), rel. Gewicht ↑, Lungenclearance eingeschränkt (ab 78. Wo); **27,8 mg/m^3^**: Lunge: Mikrogranulome (ab 26. Wo), rel. Gewicht ↑ (um 23 %, Anstieg ab 26. Wo), Foci alveolärer Bronchiolisation (ab 26. Wo)	Hesterberg et al. 1993, 1996 a
Ratte, F344N, 6 ♂	24 Mo, 0; 4,8; 15,8; 28,3 mg **MMVF11**/m^3^ (0, 41 ± 29, 153 ± 69, 246 ± 76 Fasern/ml), 6 h/d, 5 d/Wo, nur über die Nase	**4,8 mg/m^3^**: **LOAEC** Lunge: Makrophagen ↑ (Schweregrad^b)^ 2,5 von 8); **ab 15,8 mg/m^3^**: Lunge: Makrophagen ↑, Mikrogranulome, Foci alveolärer Bronchiolisation (Schweregrad^b)^ 3 von 8), Lungenclearance eingeschränkt (ab 78. Wo); **28,3 mg/m^3^**: Lunge: Mikrogranulome (ab 26. Wo), Foci (ab 26. Wo)	

^a)^ Wenn nicht anders angegeben, sind die aufgeführten Veränderungen der kontinuierlichen Variablen statistisch signifikant.

^b)^ Schweregrad nach Wagner-Einstufung (1: kein Befund, 2: nur Makrophagenansammlung, 3: Bronchiolisation oder Entzündungen, 4–8: Fibrosen unterschiedlicher Schwere; (Hesterberg et al. 1993))

AP: Alkalische Phosphatase; BALF: bronchoalveoläre Lavageflüssigkeit; d: Tag(e); h: Stunden; LDH: Laktat-Dehydrogenase; Mo: Monat; NAG: *N*-Acetyl-β-D-Glucosaminidase; n. s.: nicht signifikant; PLF: pleurale Lavageflüssigkeit; rel.: relativ; Wo: Woche(n)

##### 5.2.1.2 Hamster

Je zwölf männliche Syrische Hamster wurden vier oder zwölf Wochen lang nur über die Nase gegen 0 oder 44 mg **MMVF10a**/m^3^ (761 Fasern/ml, davon 610 WHO-Fasern/ml) vier Stunden pro Tag an fünf Tagen in der Woche exponiert und zwölf Wochen nachbeobachtet. Die GML der Fasern betrug 12,5 µm (GSD 2,5), der GMD 0,9 µm (GSD 1,6). Im eingesetzten Aerosol belief sich der Partikelanteil auf ca. 13 %. In Tabelle 10 sind die Faserbelastungen in Lunge und Pleura dargestellt. Die Fasern in der Lunge waren kürzer und dünner als im eingesetzten Aerosol. In der Pleura traten deutlich weniger Fasern auf als in der Lunge. Die Anzahl der Fasern war nach der Nachbeobachtungszeit in Lunge und Pleura deutlich reduziert. In der BALF fanden sich bereits nach vier Wochen statistisch signifikant erhöhte Lymphozytenzahlen und nach zwölf Wochen auch statistisch signifikant erhöhte Neutrophilenzahlen. Ein Anstieg der Enzymaktivität von LDH und NAG sowie der Gesamtproteinmenge wurde nicht beobachtet. Die Aktivität der Alkalischen Phosphatase war nach vier Wochen erhöht. Während die Makrophagen in der Lavage des Pleuralspaltes zu allen Untersuchungszeitpunkten in erhöhter Zahl auftraten, wurde dies bei Lymphozyten nur nach vierwöchiger Exposition beobachtet. Mesotheliale Zellreplikation (Methode BrdU-Einbau) wurde bei den Hamstern im Rippenfell nach vier Wochen und in der Pleura diaphragmatica nach zwölf Wochen Exposition nachgewiesen. Die Autoren interpretieren die Ergebnisse in der Pleura als minimale Entzündung (Bermudez et al. 2003).

In einer Inhalationsstudie wurden je 31 männliche Syrische Hamster sechs Stunden täglich an fünf Tagen pro Woche über einen Zeitraum von 13 Wochen nur über die Nase gegen **MMVF10.1** exponiert. Die Konzentrationen betrugen 0; 3,2; 16,5; 30,5; 44,5 oder 62,2 mg/m^3^. Die Konzentrationen der eingesetzten Aerosole sowie der nach Trocknung oder Veraschung in der Lunge wiedergefundenen Fasern sind in Tabelle 16 dargestellt. Die aus der Lunge gewonnenen Fasern wiesen keine signifikanten konzentrations- oder zeitabhängigen Dimensionsunterschiede auf. Die Tiere wurden sechs oder zehn Wochen nachbeobachtet. Die Faser-Lungenbelastung stieg konzentrationsabhängig an, jedoch nicht im erwarteten Verhältnis zur Aerosolkonzentration. Ab der niedrigsten Konzentration wurden erhöhte Makrophagenzahlen und Mikrogranulome in der Lunge beobachtet. Ein Schweregrad von 2,0 wurde nach der Wagner-Einstufung ermittelt und er wurde auch bei der höchsten Konzentration nicht überschritten. In der Nachbeobachtungszeit nahm die Faseranzahl in der Lunge ab. In der BALF wurden ab 16,5 mg/m^3^ eine statistisch signifikant erhöhte Zellproliferation sowie statistisch signifikant vermehrt Neutrophile und Lymphozyten festgestellt. Bei 30 mg/m^3^ wurde eine erhöhte LDH-Aktivität nachgewiesen und die Gesamtproteinmenge war statistisch signifikant erhöht. Nach zehnwöchiger Nachbeobachtung wurden diese Effekte in der BALF nicht mehr beobachtet. Die bei den beiden höchsten Konzentrationen aufgetretenen Makrophagen-Faser-Konglomerate in der Lunge sprechen für eine Überlastung der Clearance-Mechanismen. Die Clearance-Halbwertszeit der langen MMVF10.1-Fasern (Methode ^57^Co-Latex-Microspheres) betrug 14,5 Tage (Hesterberg et al. 1999).

**Tab. 16:** Faserkonzentrationen im eingesetzten Aerosol und Faserzahlen in der Lunge von Hamstern nach 13-wöchiger Exposition gegen MMVF10.1 (Hesterberg et al. 1999)

**Massenkonzentration [mg/m^3^]**	**Faserkonzentration im Aerosol** **[Fasern/ml]**		**Faserzahlen in der Lunge** **[Fasern × 10^6^/Lunge]**	
	**WHO**	**> 20 µm lang**	**WHO**	**> 20 µm lang**
3,2	36	14	0,7	0,01
16,5	206	81	6,6	0,27
30,5	316	135	7,3	0,23
44,5	552	223	13,2	0,23
62,2	714	343	12,4	0,37

Bei einer chronischen Inhalationsstudie mit je 125 männlichen Syrischen Hamstern und 140 Tieren in der Kontrollgruppe wurden die Tiere 13, 52 oder 78 Wochen (18 Monate) lang an fünf Tagen pro Woche und sechs Stunden täglich gegen 0 oder 30 mg **MMVF10a**/m^3^ nur über die Nase exponiert. Die Analyse dieser Glaswolle ergab Gehalte von 57,21 % Siliciumdioxid, 5,1 % Aluminiumoxid, 8,4 % Bortrioxid, 7,17 % Calciumoxid, 4,48 % Magnesiumoxid und 15,64 % Natriumoxid (die Gehalte wurden als Oxide angegeben, es liegen aber in der Glaswolle die Kationen vor). Nach der jeweiligen Expositionsphase erfolgte eine Nachbeobachtung bis zur 78. Woche und nach der 78-wöchigen Exposition bis zur 84. Woche. Die Exposition entsprach 323 ± 57 WHO-Fasern/ml und 151 ± 22 Fasern/ml mit einer Länge > 20 µm. Die Fasern wiesen einen GMD von 0,85 µm (GSD 1,65) und eine GML von 12,6 µm (GSD 2,4) auf. Nach 52-wöchiger Exposition hatten die Fasern in der Lunge einen GMD von 0,50 µm (GSD 1,61) und eine GML von 6,4 µm (GSD 1,9). Die Lungenbelastung mit den WHO-Fasern nahm zeitabhängig zu, jedoch nicht mit den „langen“ Fasern (Tabelle 17). Nach 78-wöchiger Exposition wurden signifikant erhöhte Fasermengen in den Lymphknoten, der Brustwand und der Pleura diaphragmatica gefunden und das relative Lungengewicht war leicht, aber nicht statistisch signifikant, angestiegen. Ab der 13. Woche wurde eine Zunahme pulmonaler Makrophagen und Mikrogranulome in der Lunge beobachtet. Zudem traten wenige Riesenzellen auf, die nach 52 Wochen jedoch nicht mehr beobachtet wurden. Nach 52 Wochen lagen minimale, aber statistisch signifikant erhöhte Fibrosen in der Pleura visceralis vor. Nach 78 Wochen war der Fibrose-Index minimal, aber nicht mehr statistisch signifikant erhöht und nach sechs Wochen Nachbeobachtung dann deutlich abgesunken, wie auch die Faserzahl in der Lunge. Die Untersuchung der BALF nach 78-wöchiger Exposition ergab eine erhöhte Anzahl an Neutrophilen. In der Lunge betrug der Schweregrad nach Wagner 2,2 nach 52-wöchiger und 2,0 nach 78-wöchiger Exposition. Nach den jeweiligen Nachbeobachtungszeiten konnte eine leichte Erholung festgestellt werden. Mesotheliale Zellproliferationen wurden in der Pleura diaphragmatica nachgewiesen. Tumoren wurden in beiden Expositionsgruppen nicht beobachtet. Nach 17 Wochen trat Mortalität aufgrund einer Enteritis/Enterotoxämie bei den Tieren auf, diese konnte durch eine Veränderung des Futters wieder eingeschränkt werden. Die Halbwertszeit von ^57^Co-markierten Latex-Microspheres betrug 28 Tage und damit war die Clearance im Vergleich zu den Kontrolltieren schneller. Die Autoren interpretieren die Daten dahingehend, dass MMVF10a nur eine reversible Entzündungsreaktion verursachte, wie sie bei jedem in die Lunge eingebrachten Partikel zu erwarten wäre (Hesterberg et al. 1997, 1999; McConnell et al. 1999).

**Tab. 17:** Lungenbelastung von Hamstern nach Exposition gegen 30 mg MMVF10a/m^3^ bei verschiedener Expositionsdauer (arithmetische Mittelwerte ± Standardabweichung) (McConnell et al. 1999)

**Faserzahl**	**Expositionsdauer [Wochen]**				
	**13 **	**26 **	**52 **	**78**	**78/6^a)^**
WHO-Fasern × 10^6^	15,4 ± 5,0	19,2 ± 5,7	31,9 ± 10,3	76,7 ± 20,5	27,8 ± 15,3
WHO-Fasern Länge > 20 µm × 10^6^	1,09 ± 0,36	0,69 ± 0,20	0,70 ± 0,47	4,60 ± 2,08	0,23 ± 0,17

^a)^ 6 Wochen Nachbeobachtung

##### 5.2.1.3 Fazit

Nach zweijähriger Exposition von Ratten gegen 3,1 mg **MMVF10**/m^3^ und 4,8 mg **MMVF11**/m^3^ wurden die Lungeneffekte nach der Wagner-Einstufung mit 2,5 bewertet, somit traten außer der Zunahme der Makrophagen auch Entzündungen oder Bronchiolisation bei einigen Tieren auf. Die Untersuchung der BALF ergab nach 13-wöchiger Exposition gegen MMVF10 bzw. MMVF10a erst bei der nächsthöheren Konzentration von 16,5 mg/m^3^ bei Ratten und Hamstern einen Anstieg an Neutrophilen, Lymphozyten, LDH-Aktivität und Gesamtprotein. Die LOAEC von **MMVF10 **und **MMVF11 **liegen damit bei 3,1 und 4,8 mg/m^3^.

#### 5.2.2 Intratracheale Aufnahme

Je acht männlichen Fischer-344-Ratten wurde einmal 0 oder 2 mg **MMVF10** oder viermal 2 mg (2 mg/Woche) intratracheal instilliert. Die Stammlösung enthielt 10 mg MMVF10/ml Kochsalzlösung. Vier oder 16 Wochen nach der letzten Gabe wurde eine bronchoalveoläre Lavage durchgeführt. Tumornekrosefaktor-α und Interleukin-1 waren nach vier Wochen bei allen exponierten Tieren in der BALF erhöht. Es wurde kein Anstieg an Neutrophilen beobachtet. Nach der Wagner-Einstufung wurden die Lungenveränderungen nach Instillation von 2 mg/Tier mit 3 (= Entzündung) und die bei 8 mg/Tier mit 4 (= geringfügige Fibrosen) bewertet (Topinka et al. 2006).

Eine Dosis von 2 mg **0,45-µm-MD-Fasern** in 2 ml Kochsalzlösung wurde fünfmal (einmal/Woche) intratracheal an weibliche Osborne-Mendel-Ratten verabreicht. Der GMD der Fasern betrug 0,4 µm und die GML 4,7 µm. Die Tiere wurden am Ende ihrer Lebenszeit untersucht. Bei drei von 22 Tieren (14 %) wurden bronchoalveoläre Metaplasien und bei sieben von 22 Tieren (32 %) Fibrosen beobachtet (Smith et al. 1987). Der Aluminiumgehalt dieser Faser wurde nicht angegeben.

#### 5.2.3 Orale Aufnahme

Hierzu liegen keine Daten vor.

#### 5.2.4 Dermale Aufnahme

Hierzu liegen keine Daten vor.

### 5.3 Wirkung auf Haut und Schleimhäute

#### 5.3.1 Haut

Hierzu liegen keine Daten vor.

#### 5.3.2 Auge

Hierzu liegen keine Daten vor.

### 5.4 Allergene Wirkung

Hierzu liegen keine Daten vor.

### 5.5 Reproduktionstoxizität

Hierzu liegen keine Daten vor.

### 5.6 Genotoxizität

#### 5.6.1 In vitro

Die Faser Manville Code 100 (**JM 100**), Länge (Median) 3,5 µm und Durchmesser (Median) 0,2 µm, führte nicht zu Mutationen bei E. coli und S. typhimurium. Schwesterchromatidaustausch-Tests mit CHO-Zellen, humanen Fibroblasten oder humanen lymphoblastoiden Zellen waren negativ (Ong et al. 1997). In CHO-Zellen wurden mit der **JM 100**-Faser klastogene Effekte im Chromosomenaberrationstest beobachtet (Sincock et al. 1982).

Die Glaswolle **JM 100**, Länge (Median) 3,5 µm und Durchmesser (Median) 0,2 µm, zeigte im Mikronukleustest in V79-Zellen konzentrationsabhängig eine Zunahme an Mikronuklei. Der nach Immunfluoreszenzfärbung beobachtete Anstieg Kinetochor-positiver Mikronuklei führte dazu, dass diese Faser als aneugen bewertet wurde. Eine größere Faser (mittlere Länge 98 µm und mittlerer Durchmesser 7,3 µm) bewirkte keinen Anstieg der Mikronuklei. Die Autoren schließen daraus, dass die Fasergröße einen Einfluss hat (Ong et al. 1997).

**JM 100-Fasern **mit einem Durchmesser von 0,1–0,2 µm bewirkten bei 2 µg/cm^2^ an SHE-Zellen Aneuploidie und eine Zunahme an Mikronuklei, Binuklei und tetraploiden Zellen. Chromosomenaberrationen waren nicht statistisch signifikant erhöht (k. w. A.; Oshimura et al. 1984). Es wurde nur eine Konzentration getestet und keine Zytotoxizitätsbestimmung durchgeführt. Im Vergleich zu den Vorgaben der OECD-Prüfrichtlinie wurden bei der Bestimmung der Chromosomenaberrationen zu wenig Metaphasen ausgezählt.

#### 5.6.2 In vivo

Hierzu liegen keine Daten für Fasern mit geringer Biobeständigkeit vor.

Je zehn männlichen Fischer-344-Ratten wurde eine einzelne Dosis von 0 oder 2 mg **MMVF10** oder viermal 2 mg (2 mg/Woche) intratracheal instilliert. Die Stammlösung enthielt 10 mg MMVF10/ml Kochsalzlösung. Vier oder 16 Wochen nach der letzten Gabe wurde eine bronchoalveoläre Lavage durchgeführt und mithilfe des Comet-Assays die so erhaltenen Zellen auf DNA-Schäden untersucht. MMVF10 induzierte bei allen exponierten Tieren einen statistisch signifikanten Anstieg (p < 0,001) von DNA-Schäden in alveolären Makrophagen und in Lungenepithelzellen vom Typ II. Im Lungengewebe wurden entzündliche Lungenveränderungen ab der niedrigsten Dosis beobachtet (siehe Abschnitt 5.2.2; Topinka et al. 2006).

Je fünf männlichen homozygoten transgenen λ-lacI F344-Ratten (Big Blue^TM^ Ratten) wurde intratracheal einmalig 0, 1 oder 2 mg **MMVF10** in Kochsalzlösung verabreicht. Eine weitere Gruppe erhielt intratracheal vier Wochen lang einmal pro Woche 0 oder 2 mg MMVF10 (insgesamt 8 mg/Tier). Die Untersuchung der Tiere erfolgte vier oder 16 Wochen nach der letzten Gabe. Im Lungengewebe wurde keine erhöhte Mutationshäufigkeit beobachtet. Im Lungengewebe der Tiere, die insgesamt 8 mg/Tier erhielten, wurden nach 16 Wochen signifikant erhöhte oxidative Schäden mit dem Marker Malondialdehyd nachgewiesen. Die Studie wurde nach OECD-Prüfrichtlinie 488 durchgeführt (Topinka et al. 2006).

### 5.7 Kanzerogenität

#### 5.7.1 Kurzzeitstudien

Eine Fasersuspension der Glaswolle **JM 100** mit Längen von ca. 1–25 µm induzierte Zelltransformationen in SHE-Zellen. Die durch Mahlen erhaltenen sehr kurzen **JM 100**-Fasern mit einer Länge von ca. 0,25–2,0 µm verursachten keine Zelltransformationen und waren deutlich geringer zytotoxisch (Hesterberg und Barrett 1984).

#### 5.7.2 Langzeitstudien

##### 5.7.2.1 Intraperitoneale Injektion

Bei der intraperitonealen Injektion wird ein lokaler Bolus an Stellen des Mesothelgewebes im Versuchstier gesetzt. Das Auftreten von Mesotheliomen gibt zuverlässig Aufschluss über das kanzerogene Potenzial von Fasern. Die zufällige Entwicklung eines Mesothelioms kann ausgeschlossen werden, da die spontane Inzidenz gering ist. Die direkte Injektion von Fasern in das Peritoneum von Ratten stellt kein physiologisches Expositionsszenario für Arbeitnehmer dar. Die daraus resultierenden Mesotheliome gelten jedoch als prädiktiv, da Mesotheliome im Peritoneum nach Inhalation von Fasern beim Menschen beobachtet werden (BAuA 2023).

Durch einmalige intraperitoneale Gabe von 0,9%iger Kochsalzlösung kam es bei Ratten in mehreren Studien nicht zu Mesotheliomen (Friemann et al. 1990; Grimm et al. 2002; Rittinghausen et al. 1991).

Nach intraperitonealer Injektion von 25 mg **0,45-µm-MD-Faser** in 0,5 ml physiologischer Kochsalzlösung bei 25 weiblichen Osborne-Mendel-Ratten konnten im Bauchraum nach 24 Monaten Fibrosen bei 13/17 Tieren (76 %) und Mesotheliome bei 8/25 Tieren (32 %) beobachtet werden. Die mittlere Lebenszeit der Ratten betrug 594 Tage (siehe Abschnitt 5.2.1 und 3.1.1, Smith et al. 1987).

Weiblichen Wistar-Ratten wurden jeweils intraperitoneal 2 ml 0,9%ige Kochsalzlösung mit Glaswolle injiziert. Bei intraperitonealer Gabe der sehr hohen Dosen von 2,7 × 10^9^ oder 5,0 × 10^9^**B-01/0.9-Fasern** (5 × 25 mg oder 10 × 25 mg/Woche) wurden bei drei bzw. vier von jeweils 40 Tieren Mesotheliome an der Bauchhaut nachgewiesen. Nach intraperitonealer Gabe von 0,4 × 10^9^ oder 1,0 × 10^9^**MMVF11-Fasern** (2 × 35 mg oder 6 × 30 mg) traten Mesotheliome bei 12 von 40 bzw. 16 von 24 Tieren auf. Die Tiere wurden nach 24 Monaten bzw. nach Lebenszeitende untersucht. Die **B-01/0.9-**Glaswolle enthielt kein Aluminium. Ca. 65 % (Masse) der **B-01/0.9-**Glaswolle besaßen eine Länge (Median) von mehr als 5 µm und mehr als 99 % einen Durchmesser (Median) von < 3 µm. Die Zusammensetzung der Glaswollen B-01, B-09 und MMVF11 sind in Tabelle 18 dargestellt. Die Gehalte wurden als Oxide angegeben, es liegen aber in der Glaswolle die Kationen vor (Roller et al. 1996).

Bernstein et al. (1996) gibt einen Aluminiumgehalt von 0,31 % der Faser B-01/09 an.

**Tab. 18:** Zusammensetzung der bei Roller et al. (1996) intraperitoneal getesteten Glaswollen in Gew.-%

**Faser**	**SiO_2_**	**B_2_O_3_**	**Na_2_O**	**Al_2_O_3_**	**FeO + Fe_2_O_3_**	**TiO_2_**	**BaO**	**K_2_O**	**CaO**	**MgO**
MMVF11	63,4	4,5	15,5	3,9	0,3	0,1	< 0,1	1,3	7,5	2,8
B-01	60,7	3,3	15,4	–	0,2	–	–	0,7	16,5	3,2
B-09	62,0	5,0	15,0	–	0,2	6,0	–	2,9	8,8	–

Nach einer intraperitonealen Verabreichung von 0 oder 1 × 10^9^**MMVF10-Fasern** in Phosphatpuffer an je 22 SPF-Ratten (k_dis_ 122,4 ng/cm^2^/h) wurden bei 13 Tieren Mesotheliome nachgewiesen. Die Tiere wurden am Ende ihrer Lebenszeit (Median 643 Tage Gesamtüberlebensrate) untersucht. Die Fasergrößen sind in Tabelle 19 dargestellt (Miller et al. 1999).

**Tab. 19:** Größen/Anzahl der intraperitoneal injizierten MMVF10-Fasern (Miller et al. 1999)

**Masse [mg]**	**Durchmesser [µm]**	**Anzahl der injizierten Fasern (× 10^6^)**					
		**Längenkategorie [µm]**					
		**> 0,4**	**> 5**	**> 8**	**> 10**	**> 15**	**> 20**
144,4	< 0,95 µm	376	314	264	236	155	119
	> 0,95 µm	665	659	598	567	506	436

Je 44–48 weiblichen Wistar-Ratten wurden die Glaswollen der Gruppe **B-1** und **B-2**, die keinen Aluminiumgehalt besaßen, intraperitoneal injiziert. Es wurden einmalig 20 mg Fasern in 2 ml Kochsalzlösung/Tier oder an drei aufeinanderfolgenden Wochen einmal wöchentlich je 20 mg Fasern in 2 ml Kochsalzlösung/Tier (insgesamt 60 mg) injiziert. In Tabelle 20 sind die Tumorinzidenzen dargestellt. Angegeben sind Mesotheliome und Sarkome der Ratten, die länger als 40 Wochen lebten. Im Vergleich mit den biobeständigeren Glaswollen (M475 und B-3) betrug die Wiederfindung in der Lunge bei diesen Glaswollen ein Jahr nach einer intratrachealen Gabe weniger als 1 %. Die Glaswollen B-1 und B-2 unterscheiden sich nicht in der Zusammensetzung, sondern nur im Durchmesser (Median) mit ca. 1,5 µm bei B-1 und 0,5 µm bei B-2 und in den Halbwertszeiten. Die Bezeichnungen K, M und L beziehen sich auf die Länge der Fasern (Halbwertszeiten siehe Abschnitt 3.1, Pott et al. 1991).

Die Daten mit intraperitonealer Gabe von 3 × 50 mg B-1K/Tier, 2 × 50 mg B-1ML/Tier und 2 × 50 mg B-2L/Tier (einmal pro Woche) an weibliche Wistar-Ratten sind ebenfalls in Tabelle 20 aufgenommen. Diese Daten sind kursiv gesetzt, da durch eine zusätzliche Lungeninfektion weniger Tiere überlebten und daher die Aussagekraft eingeschränkt ist (Pott et al. 1991).

**Tab. 20:** Tumorinzidenz nach intraperitonealer Gabe verschiedener Glaswollen an Wistar-Ratten (Pott et al. 1991)

**Glaswolle**	**Dosis**		**Fasergröße (Median) µm**		**Ratten mit Tumoren**	
	**mg**	**Faseranzahl × 10^9^**	**Länge**	**Durchmesser**	**Anzahl/gesamt**	**Mittlere Lebensdauer ** **[Wochen]**
B-1K	60	0,24	7,4	1,06	3/46	89
	*150*	*0,60*			*1/32*	*106*
B-1M	20	0,05	10,7	1,68	1/48	107
	60	0,16			1/46	75
B-1ML	*100*	*0,51*	*11,0*	*1,19*	*1/39*	*124*
B-1L	20	0,04	17,8	1,40	1/48	107
	60	0,11			5/46	109
B-2K	6,7	0,29	4,2	0,49	0/48	–
	20	0,86			0/46	–
B-2L	6,7	0,39	6,0	0,51	0/45	–
	20	1,16			2/44	109
	*100*	*5,8*	* 6,0*	*0,61*	*1/35*	* 88*

kursiv: Tiere mit Infektion

Eine zwei-, acht oder zwanzigmalige intraperitoneale Injektion von je 0 oder 2,5 × 10^8^ Fasern in je 2,5 ml 0,9%iger Kochsalzlösung wurde je 51 weiblichen Wistar-Ratten verabreicht. Das entspricht insgesamt einer Anzahl von 0,5 × 10^9^ (ca. 41–72 mg); 2 × 10^9^ (ca. 205–290 mg) oder 5 × 10^9^ Fasern (410–724 mg). Die Injektionen wurden in Abständen von einer Woche durchgeführt. Die Tiere wurden nach 123 Wochen untersucht. Die intraperitoneale Gabe von Faser M führte nicht zu einem Mesotheliom. Bei den Fasern B, P und V traten bei 2 × 10^9^ Fasern drei, vier bzw. ein Mesotheliome und bei 5 × 10^9^ Fasern 9, 8 bzw. 14 Mesotheliome am Peritoneum auf. Die Längen und Durchmesser der eingesetzten Fasern B, M, P und V sind in Tabelle 21 und die Löslichkeit in Tabelle 22 dargestellt. Die Zahlen für die Lösungsgeschwindigkeitskonstante k_dis_/SiO_2_ basieren auf dem SiO_2_-Verlust und wurden gemäß einem EURIMA-Testprotokoll analysiert. Diese Lösungsgeschwindigkeitskonstante wird laut der Autoren in keinem Regulierungskriterium verwendet (Grimm et al. 2002). Die Tabellentitel der Tabelle 2 (Grimm et al. 2002, table 2, S. 589) sind vermutlich vertauscht und werden von der Kommission anders interpretiert.

**Tab. 21:** Längen und Durchmesser der eingesetzten Fasern (Grimm et al. 2002)

**Parameter**	**Faser**			
	**B**	**M**	**P**	**V**
GMD [µm]	0,55	0,42	0,43	0,80
GML [µm]	9,27	8,26	9,60	9,90
L < 5 µm [%]	27,0	28,7	25,7	18,0
5 < L < 15 µm [%]	43,7	50,3	41,7	52,3
15 < L < 30 µm [%]	19,0	14,3	21,0	19,3
Partikel [%]	5,6	0,4	0,5	28,5

GMD: geometrischer Mittelwert Durchmesser; GML: geometrischer Mittelwert Länge; L: Länge

**Tab. 22:** Löslichkeit der eingesetzten Fasern (Grimm et al. 2002)

**Faser**		**Halbwertszeit nach intratrachealer Gabe [d]**	
	**k_dis_/SiO_2_ [ng/cm^2^/h]**	**Faser L > 20 µm**	**WHO-Faser**
B	580	4,0	17
M	103,7	5,5	8,5
P	610	12,0	21,0
V	450	n. b.	n. b.

d: Tage; k_dis_/SiO_2_: Lösungsgeschwindigkeitskonstante basierend auf SiO_2_-Verlust; L: Länge; n. b.: nicht bestimmt

Ein Vergleich der Faseranzahl von unterschiedlich biobeständigen Glaswollen, die nach intraperitonealer Gabe an Ratten zu einer Tumorinzidenz von 25 % führte, konnte zeigen, dass für die Glaswolle B-01/09 (HWZ < 40 Tage) mit 12,5 × 10^9^ mit deutlichem Abstand die höchste Anzahl Fasern benötigt wurde. Für die biobeständigeren Glaswollen **MMVF11** (HWZ 199 Tage) und **M-475** (HWZ 183 Tage) sind 0,31 × 10^9^ und 0,45 × 10^9^ Fasern angegeben. Bei den Asbestfasern Aktinolith und Krokydolith (HWZ 689 Tage) reichten 0,004 × 10^9^ bzw. 0,01 × 10^9^ Fasern für eine 25%ige Tumorinzidenz aus (AGS 2002). Die hier angegebenen Halbwertszeiten beziehen sich auf die intratracheale Instillation. Die Daten lassen also auf eine deutlich geringere kanzerogene Potenz der weniger biobeständigen Glasfasern schließen.

##### Inhalation

Je 128 männliche Fischer-344/N-Ratten wurden bis zu 24 Monate lang, an fünf Tagen pro Woche und sechs Stunden täglich, gegen 0; 3,1; 17,1 oder 27,8 mg **MMVF10**/m^3^ oder 0; 4,8; 15,8 oder 28,3 mg **MMVF11**/m^3^ exponiert. Diese gravimetrischen Konzentrationen entsprachen durchschnittlichen WHO-Faserzahlen (± SD) von 0; 29 ± 7,5; 145 ± 35 bzw. 232 ± 56 Fasern/ml für MMVF10 und 41 ± 29, 153 ± 69 bzw. 246 ± 76 Fasern/ml für MMVF11. Alle Tiere, die mindestens ein Jahr exponiert waren, wurden auf neoplastische Veränderungen geprüft. Um das Auftreten von Mesotheliomen erfassen zu können, wurden Tiere, die 24 Monate exponiert waren, erst am Ende ihrer Lebenszeit untersucht. Es wurden keine Mesotheliome beobachtet. In Tabelle 23 und 24 finden sich die Tumorinzidenzen in der Lunge (Hesterberg et al. 1993).

**Tab. 23:** Studie zur Kanzerogenität von Glaswolle MMVF10

Autor:	Hesterberg et al. 1993				
Stoff:	Glaswolle **MMVF10**				
Spezies:	**Ratte**, F344/N, 128 ♂ pro Gruppe				
Applikation:	Inhalation				
Konzentration:	0; 3,1; 17,1; 27,8 mg/m^3^				
Dauer:	12–24 Mo, 5 d/Wo, 6 h/d				
Toxizität:	ab 3,1 mg/m^3^: Entzündungszeichen in der Lunge (siehe Abschnitt 5.2.1)				
		**Konzentration [mg/m^3^]**			
		**0**	**3,1 **	**17,1 **	**27,8**
Überlebende	♂	123/128 (96 %)	117/128 (91 %)	118/128 (92 %)	119/128 (93 %)
**Tumoren **					
**Lunge:**					
Adenome	♂	3/123 (2,4 %)	0/117 (0 %)	1/118 (0 %)	6/119 (5,0 %)
Karzinome	♂	1/123 (0,8 %)	0/117 (0 %)	0/118 (0 %)	1/119 (0,8 %)

**Tab. 24:** Studie zur Kanzerogenität von Glaswolle MMVF11

Autor:	Hesterberg et al. 1993				
Stoff:	Glaswolle **MMVF11**				
Spezies:	**Ratte**, F344/N, 128 ♂ pro Gruppe				
Applikation:	Inhalation				
Konzentration:	0; 4,8; 15,8; 28,3 mg/m^3^				
Dauer:	12–24 Mo, 5 d/Wo, 6 h/d				
Toxizität:	ab 4,8 mg/m^3^: Entzündungszeichen in der Lunge (siehe Abschnitt 5.2.1)				
		**Konzentration [mg/m^3^]**			
		**0**	**4,8 **	**15,8 **	**28,3**
Überlebende	♂	123/128 (96 %)	118/128 (92 %)	120/128 (94 %)	112/128 (88 %)
**Tumoren**					
**Lunge:**					
Adenome	♂	3/123 (2,4 %)	3/118 (2,5 %)	6/120 (5,0 %)	3/112 (2,7 %)
Karzinome	♂	1/123 (0,8 %)	1/118 (0,9 %)	3/120 (2,5 %)	0/112 (0 %)

d: Tag(e); h: Stunden; Mo: Monate; Wo: Woche

Je 24 männliche und weibliche Han-Wistar-Ratten pro Gruppe wurden 12 oder 24 Monate lang gegen 0 oder 5,2 bzw. 4,96 mg **SG Glaswolle**/m^3^ ganzkörperexponiert. Nach 24-monatiger Exposition zeigte ein Tier ein Plattenepithelkarzinom in der Lunge. Bei vier weiteren Tieren traten Tumoren im Magen-Darm-Trakt auf, die nicht auf die Faserexposition zurückgeführt wurden (siehe Abschnitt 5.2.1, Le Bouffant et al. 1987).

Je 130 bzw. 121 männliche Fischer-344N-Ratten wurden zwei Jahre lang, an sechs Stunden pro Tag und fünf Tagen in der Woche, gegen Glaswolle **X607** in Konzentrationen von 0 oder 30 mg/m^3^ nur über die Nase exponiert. Tabelle 25 zeigt die Inzidenzen der aufgetretenen Adenome, Karzinome und Hyperplasien in der Lunge. Mesotheliome wurden nicht beobachtet (siehe Abschnitt 3.1 und 5.2.1, Hesterberg et al. 1998).

**Tab. 25:** Studie zur Kanzerogenität von Glaswolle X607

Autor:	Hesterberg et al. 1998		
Stoff:	Glaswolle **X607**		
Spezies:	**Ratte**, F344/N, 130/121 ♂ pro Gruppe		
Applikation:	Inhalation		
Konzentration:	0, 30 mg/m^3^		
Dauer:	24 Mo, 5 d/Wo, 6 h/d		
Toxizität:	30 mg/m^3^: Entzündungszeichen Lunge (siehe Abschnitt 5.2.1)		
		**Konzentration [mg/m^3^]**	
		**0**	**30**
Überlebende	♂	k. A./130	k. A./121
**Tumoren und Präneoplasien**			
**Lunge:**			
Adenome	♂	2/130 (1,5 %)	1/121 (0,8 %)
Karzinome	♂	0/130 (0 %)	1/121 (0,8 %)
Hyperplasien, bronchoalveolär	♂	5/130 (3,8 %)	6/121 (4,9 %)

d: Tag(e); h: Stunden; k. A.: keine Angabe; Mo: Monate; Wo: Woche

In einer Inhalationsstudie mit je 125–140 männlichen Syrischen Hamstern wurden die Tiere 18 Monate lang an fünf Tagen die Woche und sechs Stunden täglich gegen 0 oder 30 mg **MMVF10a**/m^3^ exponiert. Es traten keine pulmonalen oder pleuralen Tumoren auf (siehe Abschnitt 5.2.1; Hesterberg et al. 1997, 1999; McConnell et al. 1999).

#### 5.7.3 Fazit

Ein Vergleich der Tumorhäufigkeit nach Inhalation und dem Auftreten von Mesotheliomen nach intraperitonealer Gabe zeigt, dass bei intraperitonealer Gabe deutlich mehr Mesotheliome entstehen als nach Inhalation. Die intraperitoneale Verabreichung stellt damit eine sehr empfindliche Methode dar. Bei Glaswollen (HWZ < 40 d) treten vereinzelt Mesotheliome nur nach intraperitonealer Gabe sehr hoher Faserzahlen (> 1 × 10^9^/Tier) auf, während bei MMVF11 mit einer intratracheal ermittelten Halbwertszeit von 199 Tagen nach intraperitonealer Gabe von 0,5 × 10^9^ Fasern/Tier mehr als die Hälfte der Tiere Mesotheliome aufwies.

## 6 Bewertung

Kritische Effekte sind entzündliche Prozesse im Atemtrakt. Eine kanzerogene Wirkung ist bei Exposition gegen sehr hohe Dosen und Überlastung der Clearance nicht auszuschließen.

**MAK-Wert. **Es liegen keine ausreichenden epidemiologischen Daten zur Bewertung vor (siehe Abschnitt 4; Hartwig und MAK Commission 2018).

Bei einer geringen Biobeständigkeit von Glaswolle, die keine typische Fasertoxizität mehr erwarten lässt, kann bei Einhaltung eines Grenzwertes keine kanzerogene Wirkung auftreten. Für Glaswolle mit einer Halbwertszeit nach intratrachealer Gabe von unter 40 Tagen wird dies als hinreichend belegt angesehen, da die typische Faserkanzerogenität im sehr sensitiven Test auf Mesotheliome nach intraperitonealer Gabe bei dieser Glaswolle erst nach Verabreichung sehr hoher Fasermengen zum Auftreten einzelner Mesotheliome führte.

Da immer ein Anteil granulärer Partikel in den in Inhalationsstudien eingesetzten Aerosolen vorhanden ist und auch bei der Produktion und Bearbeitung von Glaswolle granuläre Partikel am Arbeitsplatz entstehen, ist der MAK-Wert auf die Masse faserförmiger und partikulärer Bestandteile bezogen und nicht auf die Faseranzahl.

Da keine Inhalationsstudien mit Glaswollen vorliegen, die nach intratrachealer Gabe an Ratten eine Halbwertszeit von weniger als 40 Tagen aufweisen, werden Inhalationsstudien der beständigeren Glaswollen MMVF10 und MMVF11 herangezogen. Nach zweijähriger Exposition gegen 3,1 mg MMVF10/m^3^ und 4,8 mg MMVF11/m^3^ wurden Zunahmen an Makrophagen in der Lunge beobachtet. Nach der Wagner-Einstufung wurden die Effekte mit 2,5 bewertet, somit traten außer der Zunahme der Makrophagen auch noch geringe entzündliche Effekte auf. Die Lungenclearance war bereits bei Exposition gegen 3,1 mg MMVF10/m^3^ eingeschränkt (Hesterberg et al. 1993). Die Untersuchung der BALF ergab nach 13-wöchiger Exposition gegen MMVF10 erst bei der nächsthöheren Konzentration von 16,5 mg/m^3^ einen statistisch signifikanten Anstieg an Neutrophilen, Lymphozyten, LDH-Aktivität und Gesamtprotein (Hesterberg et al. 1996 a). Die LOAEC für MMVF10 liegt damit bei 3,1 mg/m^3^ und für MMVF11 bei 4,8 mg/m^3^. Die bei dieser Konzentration aufgetretenen Effekte werden als gering betrachtet, sodass ausgehend von dieser LOAEC auf eine NAEC von 1 mg/m^3^ für MMVF10 und 1,6 mg/m^3^ für MMVF11 (LOAEC/3) geschlossen werden kann.

In der Studie von Nielsen und Koponen (2018) werden toxikokinetische Unterschiede zwischen Ratte und Mensch beschrieben und Faktoren ermittelt, die die unterschiedliche Belastung in der Lunge widerspiegeln. Ausgehend von einem Faser-Durchmesser von 1 µm werden bei gleicher äußerer Konzentration und nasaler Atmung dreimal so viel Fasern pro cm^2^ Lunge beim Menschen wie bei der Ratte deponiert.

Bei schwer löslichen Substanzen ergibt sich ein Faktor 6,7 für die Makrophagen-bedingte Clearance aus den von der Kommission verwendeten speziesspezifischen Clearancehalbwertszeiten im Alveolarbereich mit 60 Tagen (Ratte) bzw. 400 Tagen (Mensch) (Hartwig 2012). Um die Auflösung der biolöslichen Fasern (Halbwertszeit < 40 Tage) in der Lunge als zusätzlichen Eliminationsmechanismus zu der Makrophagenclearance zu berücksichtigen, wurde statt Faktor 6,7 mit einem reduzierten Faktor 3 bei den speziesspezifischen Clearancehalbwertszeiten (Ratte/Mensch) extrapoliert.

Nach Anwendung der Faktoren ergibt sich für die Faser MMVF10 ein Wert von 0,111 mg/m^3^ und für MMVF11 ein Wert von 0,177 mg/m^3^.

1 mg/m^3^ : 3 (Deposition) : 3 (Clearance) = 0,111 mg/m^3^

1,6 mg/m^3^ : 3 (Deposition) : 3 (Clearance) = 0,177 mg/m^3^

Ein zweiter Berechnungsweg der HEC benötigt die Depositionsfraktionen der Fasern, die nach dem MPPD-Modell (Version 3.04, ARA 2015) ermittelt werden. Die Parameter sind in Tabelle 26 und 27 aufgelistet.

**Tab. 26:** Parameter für die Fasern MMVF10 und MMVF11 (Hesterberg et al. 1993) im MPPD-Modell

**Parameter**	**MMVF10**	**MMVF11**
Durchmesser (Median)	1,26 µm	0,69 µm
GSD Durchmesser	1,81	2,10
GSD Länge	2,0	2,2
Aspect ratio^a)^	10,4	19,9
Dichte	2,5 g/cm^3^	2,5 g/cm^3^
Aerosol-Konzentration	3 mg/m^3^	4,8 mg/m^3^
Inhalierbarkeit	ja	ja

^a)^ Verhältnis von Länge zu Durchmesser, manuell errechnet

**Tab. 27:** Verwendete Parameter für Ratte und Mensch im MPPD-Modell

**Parameter**	**Ratte**	**Mensch**
Modell	semi-symmetric Long Evans	Yeh-Schum 5 Lobe
Atmung	nasal	nasal
Atemfrequenz	102/min (default)	20/min
Atemzugvolumen	2,1 ml (default)	1040 ml

Die hier eingesetzten Werte für das Atemvolumen des Menschen entsprechen 10 m^3^/8 h. Alle weiteren Parameter wurden auf Defaultwerten belassen.

Es ergaben sich folgende Depositionsfraktionen (Tabelle 28):

**Tab. 28:** Mit dem MPPD-Modell berechnete Depositionsfraktionen für Mensch und Ratte

**Faser, Spezies**	**Tracheobronchial (TB)**	**Alveolär**	**TB + Alveolär**
**MMVF10**			
Lungenlappen, human	0,0052	0,0034	0,0086
Ratte	3,659 × 10^-4^	1,086 × 10^-4^	0,0004745
**MMVF11**			
Lungenlappen, human	0,0050	0,0030	0,008
Ratte	0,0014	2,315 × 10^-4^	0,0016315

Das Verhältnis der Depositionsfraktionen (tracheobronchial plus alveolär) Ratte/Mensch beträgt demnach für MMVF10:

0,0004745/0,0086 = 0,055

und für MMVF11:

0,0016315/0,008 = 0,2

Für die HEC-Berechnung für den Arbeitsplatz wird folgende Formel verwendet:

HEC = 0,008 (Expositionszeit- und Atemvoluminaverhältnisse Tier/Mensch (FoBiG 2011)) × 192,7 (Verhältnis Lungenoberfläche Mensch/Tier (Hartwig 2012)) × Depositionsfraktion Tier / Depositionsfraktion Mensch / 3 (Clearanceunterschied Mensch/Ratte) × LOAEC Tier / 3

Damit ergeben sich folgende Konzentrationen für den Arbeitsplatz:

MMVF10 (Durchmesser 1,26 µm): 0,008 × 192,7 × 0,055 / 3 × 3,1 mg/m^3 ^/ 3 = 0,029 mg/m^3^

MMVF11 (Durchmesser 0,69 µm): 0,008 × 192,7 × 0,2 / 3 × 4,8 mg/m^3 ^/ 3 = 0,164 mg/m^3^

Nach Aussage von Nielsen und Koponen (2018) liegen am Arbeitsplatz Glaswollen mit Durchmesser von 1 µm vor. Der Durchschnitt aus den Durchmessern von MMVF10 und MMVF11 ist etwa 1 µm und der Mittelwert aus beiden Konzentrationen von 29 und 164 µg/m^3^ ist 97 µg/m^3^.

In einer Auswertung von Daten zu Lungenfibrose und Lungenkrebs durch Asbest wurde bei gleicher Faserzahl pro Gramm Lungengewebe eine ähnliche Empfindlichkeit von Ratten und Menschen abgeleitet, es liegen aber auch Daten vor, nach denen der Mensch empfindlicher ist (Maxim und McConnell 2001; Nielsen und Koponen 2018). Der Mechanismus der Toxizität von Asbest muss aber nicht mit dem von Glaswolle übereinstimmen. Für die Glaswollen (HWZ < 40 d) wird, ähnlich wie bei GBS, die Überlastung der Lungenclearance für die Toxizität verantwortlich gemacht. Daher wird, wie bei GBS, für die Übertragung der Daten des Tierversuchs auf den Menschen kein zusätzlicher Faktor berücksichtigt.

Damit kann aus diesen Studien nach dem „Preferred Value Approach“ ein MAK-Wert von 0,1 mg/m^3^ für die A-Fraktion von Glaswolle (faserförmige und granuläre Bestandteile) mit einer Halbwertszeit von unter 40 Tagen (nach intratrachealer Verabreichung der WHO-Fasern) abgeleitet werden.

Für die hier eingesetzte Glaswolle MMVF11 wurde eine Halbwertszeit nach intratrachealer Gabe von 199 Tagen ermittelt (Abschnitt 3.1.1). Damit liegt die Halbwertszeit dieser Faser deutlich über dem Kriterium von < 40 Tagen nach intratrachealer Gabe für Glaswollen, für die der hier abgeleitete Grenzwert gilt. Die Faser MMVF10 zeigte nach Inhalation einen längeren Verbleib in der Lunge als MMVF11. Aufgrund der höheren Biobeständigkeit der Fasern MMVF10 und MMVF11 ist diese MAK-Wert-Ableitung als Worst-Case-Betrachtung anzusehen.

Sofern eine spezifische Toxizität anderer Stoffe (insbesondere Aluminium) beteiligt ist, muss diese gesondert berücksichtigt werden.

Unter der Annahme eines maximalen Aluminiumanteils in der Glaswolle von 25 % ergibt sich bezogen auf den MAK-Wert von 0,1 mg/m^3^ eine Luftkonzentration von 0,025 mg Al/m^3^. Diese Aluminiumkonzentration in der Luft liegt unter dem MAK-Wert von Aluminium und seinen schwerlöslichen Verbindungen von 0,05 mg Al/m^3^ für die A-Fraktion (Hartwig und MAK Commission 2025).

**Spitzenbegrenzung. **Der kritische Effekt von Glaswolle (HWZ < 40 d) ist die Lungentoxizität nach längerfristiger Applikation. Sie wird daher in die Spitzenbegrenzungs-Kategorie II eingestuft. Bei Inhalation ist wie bei intratrachealer Gabe eine deutlich längere Eliminationshalbwertszeit als acht Stunden anzunehmen, daher wird als Überschreitungsfaktor 8 festgelegt.

**Fruchtschädigende Wirkung. **Es fehlen Studien der hier betrachteten Glaswolle zur pränatalen Entwicklungstoxizität.

Die Glaswollen (HWZ < 40 d) werden daher der Schwangerschaftsgruppe D zugeordnet. Es gibt keine Hinweise für einen Verdacht auf eine entwicklungstoxische Wirkung.

**Krebserzeugende Wirkung. **Die kanzerogene Wirkung von Fasern aufgrund ihrer spezifischen Geometrie wurde für anorganische Faserstäube bereits bewertet und sie wurden in die Kanzerogenitäts-Kategorie 2 eingestuft (Hartwig und MAK Commission 2018).

Bei hinreichender Biolöslichkeit kann bei Glaswolle davon ausgegangen werden, dass die kanzerogene Wirkung aufgrund der Fasertoxizität bei Einhaltung des MAK-Wertes nicht auftritt. Dies trifft für Glaswollen zu, die nach intratrachealer Instillation in der Rattenlunge eine Halbwertszeit von weniger als 40 Tagen aufweisen.

Glaswollen (HWZ < 40 d) werden in die Kanzerogenitäts-Kategorie 4 eingestuft. Bei Einhaltung des MAK-Wertes ist kein Beitrag zum Krebsrisiko beim Menschen zu erwarten.

**Keimzellmutagene Wirkung. **Nach bis zu vierwöchiger intratrachealer Gabe an Ratten wurden DNA-Schäden in der Lunge bei gleichzeitiger Zytotoxizität nachgewiesen, jedoch keine Mutagenität bei transgenen Tieren. Untersuchungen an Keimzellen liegen nicht vor. Es erfolgt keine Einstufung in eine Kategorie für Keimzellmutagenität.

**Hautresorption. **Zur dermalen Aufnahme liegen keine Studien vor. Da es sich um mikroskalige Feststoffe handelt, von denen anzunehmen ist, dass sie die Hautbarriere nicht überwinden können und deren systemische Wirkungen nicht im Vordergrund stehen, ist ein relevanter Beitrag der Hautexposition zur systemischen Toxizität unwahrscheinlich. Daher wird Glaswolle nicht mit „H“ markiert.

**Sensibilisierende Wirkung. **Es liegen keine Daten zur sensibilisierenden Wirkung von Glaswolle (HWZ < 40 d) vor.

Glaswolle (HWZ < 40 d) wird weder mit „Sh“, noch mit „Sa“ markiert.
